# Unconventional MAPK-GSK-3β Pathway Behind Atypical Epithelial-Mesenchymal Transition In Hepatocellular Carcinoma

**DOI:** 10.1038/s41598-017-09179-0

**Published:** 2017-08-18

**Authors:** Sana Parveen, Dhiviya Vedagiri, Hitha Gopalan Nair, Haripriya Parthasarathy, Krishnan Harinivas Harshan

**Affiliations:** CSIR-Centre for Cellular and Molecular Biology, Hyderabad, 500007 India

## Abstract

We recently reported an atypical epithelial mesenchymal transition (EMT) in human hepatoma cell culture Huh7.5, which was non-responsive to the canonical EMT-transcription factors. Here we characterize major pathways regulating this atypical EMT through whole genome transcriptome profiling and molecular analysis, and identify a unique regulation of EMT by GSK-3β. Our analysis reveals remarkable suppression of several key liver-specific markers in Huh7.5M cells indicating that EMT not only changes the epithelial properties, but alters the characteristics associated with hepatocytes as well. One key finding of this study is that GSK-3β, a known antagonist to β-Catenin signaling and a major pro-apoptotic regulator, is critical for the maintenance of EMT in Huh7.5M cells as its inhibition reversed EMT. Importantly, through these studies we identify that maintenance of EMT by GSK-3β in Huh7.5M is regulated by p38MAPK and ERK1/2 that has not been reported elsewhere and is distinct from another metastatic non-hepatic cell line MDA-MB-231. These data showcase the existence of non-canonical mechanisms behind EMT. The atypicalness of this system underlines the existence of tremendous diversity in cancer-EMT and warrants the necessity to take a measured approach while dealing with metastasis and cancer drug resistance.

## Introduction

Liver cancer is a major healthcare concern worldwide with hepatocellular carcinoma (HCC) being the most frequent form. According to recent reports from International Agency for Cancer Research, World Health Organization, HCC was listed as one of the five commonest and the second most lethal cancer in 2012^1^. The most frequent causative agents of HCC are Hepatitis B and C viruses (HBV and HCV)^2^. Combined, these two viruses are estimated to have infected over 400 million people worldwide. A significant part of HBV and HCV chronic infections progress to fibrosis, liver cirrhosis and then to HCC. While new HBV infections are controlled by very effective vaccine, HCV spread continues alarmingly in the absence of a vaccine.

Metastasis is the major cause of cancer related deaths in solid cancers^3, 4^. During metastasis, individual or clusters of cells leave the primary tumor, enter the vasculature and migrate to foreign sites in order to develop secondary tumors. A major hurdle in this process is the strong adherence of the epithelial cells in carcinomas. They overcome this obstacle through a process called epithelial to mesenchymal transition (EMT)^5–7^. EMT, through which epithelial cells shed many of their characteristic properties while adopting several mesenchymal features, equips them with migratory and invasive competence and drug resistance. EMT results in an overhaul of signaling, metabolic, transcriptional, cytoskeletal, membranous and extracellular landscape of the cell^8–10^. Even though it is generally accepted that EMT provides cancer cells migratory and invasive advantage, and multiple drug resistance, a couple of recent studies argue that EMT only contributes to drug resistance^11, 12^.

Members of Snail, Zeb and Twist family transcription factors (EMT-TFs) have been implicated in the transcriptional reprogramming that supports EMT^5, 7, 13, 14^. This is achieved through transcriptional repression of key epithelial markers such as E-Cadherin and Claudin1 while simultaneously activating mesenchymal markers including Vimentin, N-Cadherin and β-Catenin. This results in remarkable changes in every aspect of the cellular identity, ranging from their size and shape to metabolic preferences and evasion of immune surveillance.

TGFβ/BMP and Wnt/β-Catenin signal pathways have been commonly implicated in regulating EMT^15–22^. TGFβ signals through TGFβ receptor-SMAD2/SMAD3-SMAD4 axis to regulate transcription of EMT-TFs. BMP signaling follows similar mode, but uses SMAD1/5/8 in place of SMAD2/3 of TGFβ signaling. Independently, TGFβ signaling can activate MAPK pathway and regulate transcription. Wnt/β-Catenin pathway deeply influences many facets of cell cycle and function^17–19^. β-Catenin, a major bridge between cadherins and cytoskeletal elements, is subject to constant degradation mediated by GSK-3β in the absence of Wnt signaling. Upon activation by Wnt, Frizzled (Fz) receptors inhibit GSK-3β, thereby stabilizing β-Catenin. This results in the translocation of β-Catenin to nucleus, where it interacts with TCF/LEF transcription factors to activate transcription of β-Catenin regulated genes. In addition to these two major pathways, NF-κB seems to be essential for induction and maintenance of EMT since inhibition of NF-κB results in MET in breast cancer model^23^.

We recently characterized an atypical EMT in HCC cultures^24^. Huh7.5M cells with mesenchymal characteristics were generated from the epithelial Huh7.5 cells. Huh7.5:Huh7.5M EMT system was non-responsive to canonical EMT-TFs. Ectopic expression of EMT-TFs in Huh7.5 cells did neither drive EMT, nor did Slug depletion revert EMT phenotype in Huh7.5M cells. Whole genome transcription profiling is a powerful tool to gain valuable details on critical molecules and pathways reprogrammed during EMT. Various studies have reported transcriptional reprogramming behind EMT in cultured cells or from clinical samples^25–27^. Metadata generated from various such curated datasets identified a core gene set in EMT programs^28^. We performed microarray based transcriptome analysis of Huh7.5 and Huh7.5M cells in order to gain insight into transcriptional reprogramming behind this atypical EMT and to identify novel molecules involved in it. We identify in this study that β-Catenin signaling is phenomenally dysregulated during EMT. GSK-3β was critical in the maintenance of mesenchymal markers in Huh7.5M cells as its inhibition reverted EMT features. We also demonstrate that GSK-3β is in turn regulated by p38MAPK and ERK1/2 through a novel regulation. In addition to this pathway, BMP and NF-κB pathways were also highly dysregulated. Another important finding from our study is the loss of liver characteristics in Huh7.5M cells upon EMT. We also identify a large set of novel transcription factors, cell surface receptors, cytoskeletal molecules and regulators of various metabolic pathways in this study.

## Results

### Expression profiling identifies phenomenal transcriptional reprogramming in Huh7.5:Huh7.5M system

**Figure 1:**
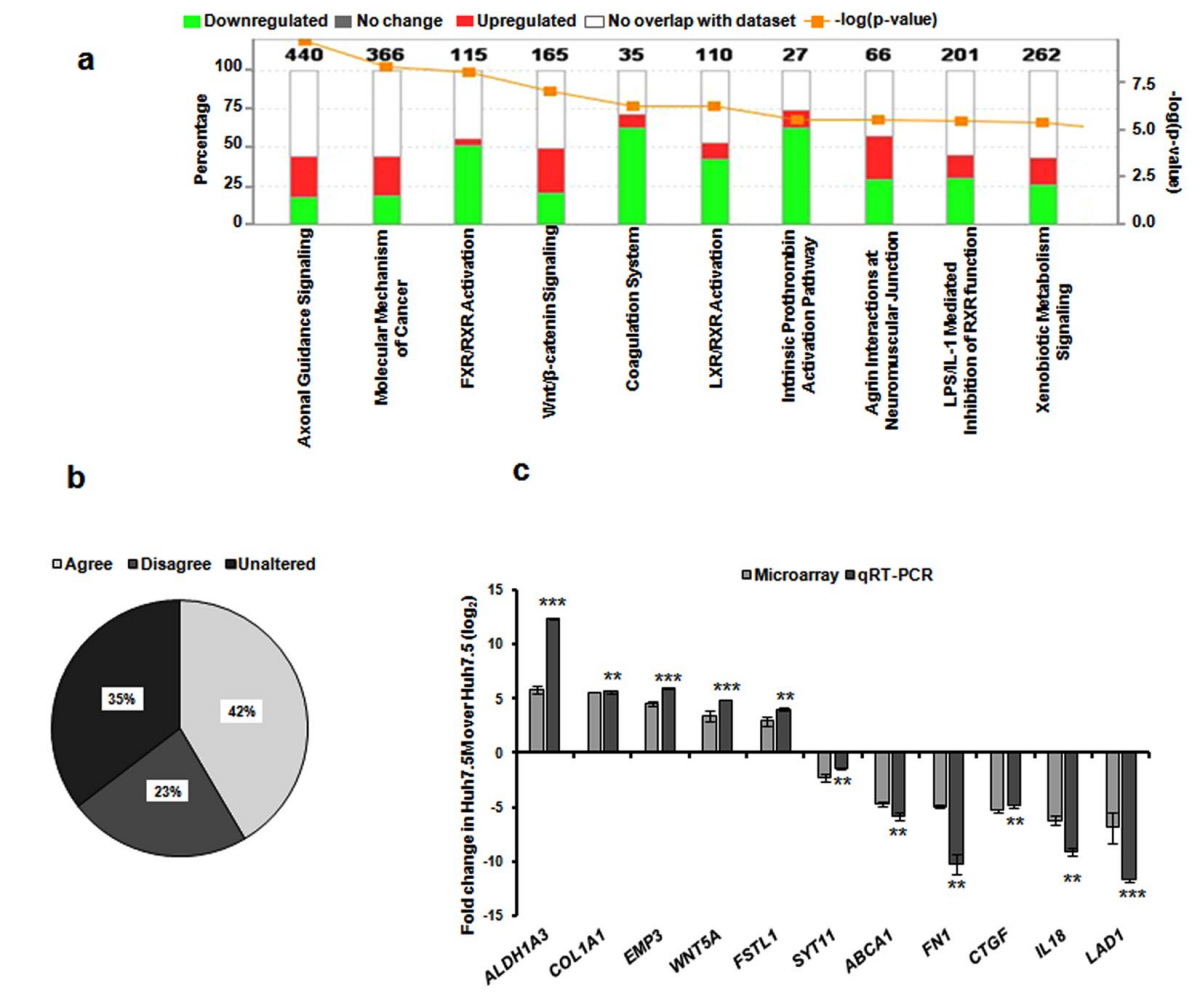
Analysis of gene expression profiles between Huh7.5 and Huh7.5M cells. (**a**) Major pathways regulated during EMT in Huh7.5:Huh7.5M system. Gene expression changes were analyzed by IPA. Red shades represent the genes upregulated in Huh7.5M cells while the green shades represent those downregulated. Numbers at the top of each histogram represents the total number of genes classified in the respective category. Left Y-axis represents the percentage of genes in the respective category that underwent regulation. Right Y axis corresponds to the –log of the *P-* value generated by right tailed Fisher’s exact test. (**b**) Comparison of EMT core gene expression between in Huh7.5:Huh7.5M system and that generated by Groger *et al*. Gene expression profiles of Huh7.5 and Huh7.5M cells were used for this comparison. “Agree” corresponds to the genes that underwent changes as suggested by the reference list; “Disagree” has those genes that were regulated in disagreement with the reference list; and “Unaltered” suggests that these genes were not detected, unlike in the reference list. (**c**) Validation of microarray results of eleven genes from EMT core list by qRT-PCR. Fold change in Huh7.5M cells over Huh7.5 cells from microarray and qRT-PCR experiments are represented. *, ** and *** indicate statistical significance of qRT-PCR data represented as *P*-values < 0.05, < 0.005 and < 0.0005 respectively.

We previously reported an atypical EMT in Huh7.5 cells that resulted in generation of Huh7.5M mesenchymal cells through geneticin resistance^24^. The atypicalness was attributed to two key features: (i) inability of canonical EMT-TFs to induce EMT in Huh7.5 and (ii) concurrent expression of epithelial markers and EMT-TFs in Huh7.5. To access deeper insight into this atypical EMT, we undertook whole genome expression profiling by microarray. Remarkably large numbers of genes (6765) were differentially expressed having average fold change ≥(log_2_ 1) and ≤(log_2_ (−1)) between Huh7.5 and Huh7.5M cells. EMT resulted in transcriptional activation of 3442 genes in Huh7.5M cells with concurrent suppression of 3323 genes. Next, we analyzed the data using IPA that indicated many differentially regulated functional groups as a consequence to EMT (Fig. 1a). Significantly, major pathways that regulate cancer progression, critical molecules in the maintenance of liver function and identity, those taking part in coagulation and components of Wnt/β-Catenin pathways were considerably regulated during EMT (Fig. 1a). Key upstream regulators identified by IPA included *HNF4A*, *HNF1A*, *PKD1, CTNNB1* and *MGEA5* (Table 1). Several genes involved in cellular assembly, functions, morphology, growth, proliferation and those contributing to disease conditions were targets of EMT. Several genes participating in embryonic and organismal development also underwent high degrees of regulation during EMT (Table 1).

**Table 1:** Top 5 Upstream Regulators, Disease and Bio functions modulated during EMT in Huh7.5:Huh7.5M cells, as predicted by IPA.

**I. Top Upstream Regulators**		
**Upstream Regulator**	***P-*** **value of overlap**	**Predicted Activation**
*HNF4A*	1.04*10^−9^	Inhibited
*HNF1A*	6.83*10^−9^	Inhibited
*PKD1*	6.98*10^−9^	Inhibited
*CTNNB1*	3.84*10^−7^	Activated
*MGEA5*	3.32*10^−5^	Inhibited
**II. Top Disease and Bio functions**		
**A. Diseases and Disorders**		
**Name**	***P*** **-value range**	**Number of molecules**
Organismal injury and abnormalities	1.16*10^−4^–5.12*10^−24^	1651
Connective tissue disorder	1.14*10^−4^–6.65*10^−21^	524
Skeletal and muscular disorder	1.14*10^−4^–6.65*10^−21^	622
Neurological disease	6.00*10^−5^–2.81*10^−18^	674
Developmental disorder	9.754*10^−5^–4.11*10^−17^	732
**B. Molecular and cellular functions**		
**Name**	***P-*** **value range**	**Number of molecules**
Cellular assembly and organization	1.32*10^−4^–1.26*10^−20^	611
Cellular function and maintenance	1.32*10^−4^–1.26*10^−20^	1097
Cellular development	1.33*10^−4^–1.90*10^−20^	1265
Cellular morphology	1.13*10^−4^–5.80*10^−20^	1128
Cellular growth and proliferation	5.70*10^−4^–1.32*10^−19^	1432
**C. Physiological system development and function**		
**Name**	***P-*** **value range**	**Number of molecules**
Embryonic development	1.29*10^−4^–1.40*10^−28^	1286
Organismal development	1.29*10^−4^–1.40*10^−28^	1860
Organismal survival	3.22*10^−4^–1.78*10^−23^	1291
Connective tissue development and function	1.25*10^−4^–1.10*10^−22^	858
Skeletal and muscular development and function	1.24*10^−4^–1.10*10^−22^	805

Various studies have reported expression profiles from EMT. Groger *et al*. performed metadata analysis of 18 independent GES of EMT and subsequently formulated a core list of 130 genes that are exclusively associated with the process^28^. We compared our dataset with this EMT core-gene list. Expression pattern of 54 genes from Huh7.5:Huh7.5M system was in agreement with the core list while 30 genes differed (Fig. 1b and Supplementary Table S1). 46 genes from the core list were not identified in our analysis. On the whole, only 42% of the core genes of Huh7.5:Huh7.5M were in agreement with EMT core gene list underlining the complexity involved in the mechanism of this EMT. Microarray profiles of select set of genes from this core list were further validated by qRT-PCR (Fig. 1c). Out of the genes that differed, *FN1* assumes significance as a major mesenchymal marker that is critical for intravasation^8^.

### Regulation of transcription mediators during EMT

Many transcription factors have been associated with EMT as regulators and contributors^29, 30^. Although canonical EMT-TFs play a critical role in driving EMT, multilayered complexity exists in reprogramming the network. 857 such genes were regulated during EMT in Huh7.5:Huh7.5M, as revealed by whole genome expression profiling. Out of this, 476 genes showed activation in Huh7.5M cells. This phenomenal regulation is in sync with the activation of *HDAC 1*, 6 and 8 in Huh7.5M that we reported previously^24^.

Zinc finger (ZNF) family, an important family of transcription factors, is involved in transcription of myriad sets of genes. A number of ZNF proteins are implicated in EMT^31^. In our analysis, 176 ZNF genes were shown to be differentially expressed during EMT in Huh7.5:Huh7.5M system with 74 being upregulated and 102 being downregulated in Huh7.5M cells (Supplementary Table S2). The list includes many ZNFs that have not previously been associated with EMT. Another important family of transcription factors shown to modulate EMT, the homeobox (Hox) family^32^, was also significantly regulated in Huh7.5M EMT. Twenty four Hox genes were activated in Huh7.5M cells, indicating their significant involvement (Supplementary Table S2). A significant number of forkhead family of transcription factor (FOX) genes with potential contribution to EMT^33^ were regulated in Huh7.5:Huh7.5M cells. Out of this, FOXF1 and FOXC1 were overexpressed, and FOXA1 and FOXA2 were downregulated in Huh7.5M, consistent with previous reports^34–36^. Supplementary Table S2 provides a list of FOX genes found in Huh7.5:Huh7.5M system.

### Extracellular matrix and Integrin Signaling in EMT

**Figure 2:**
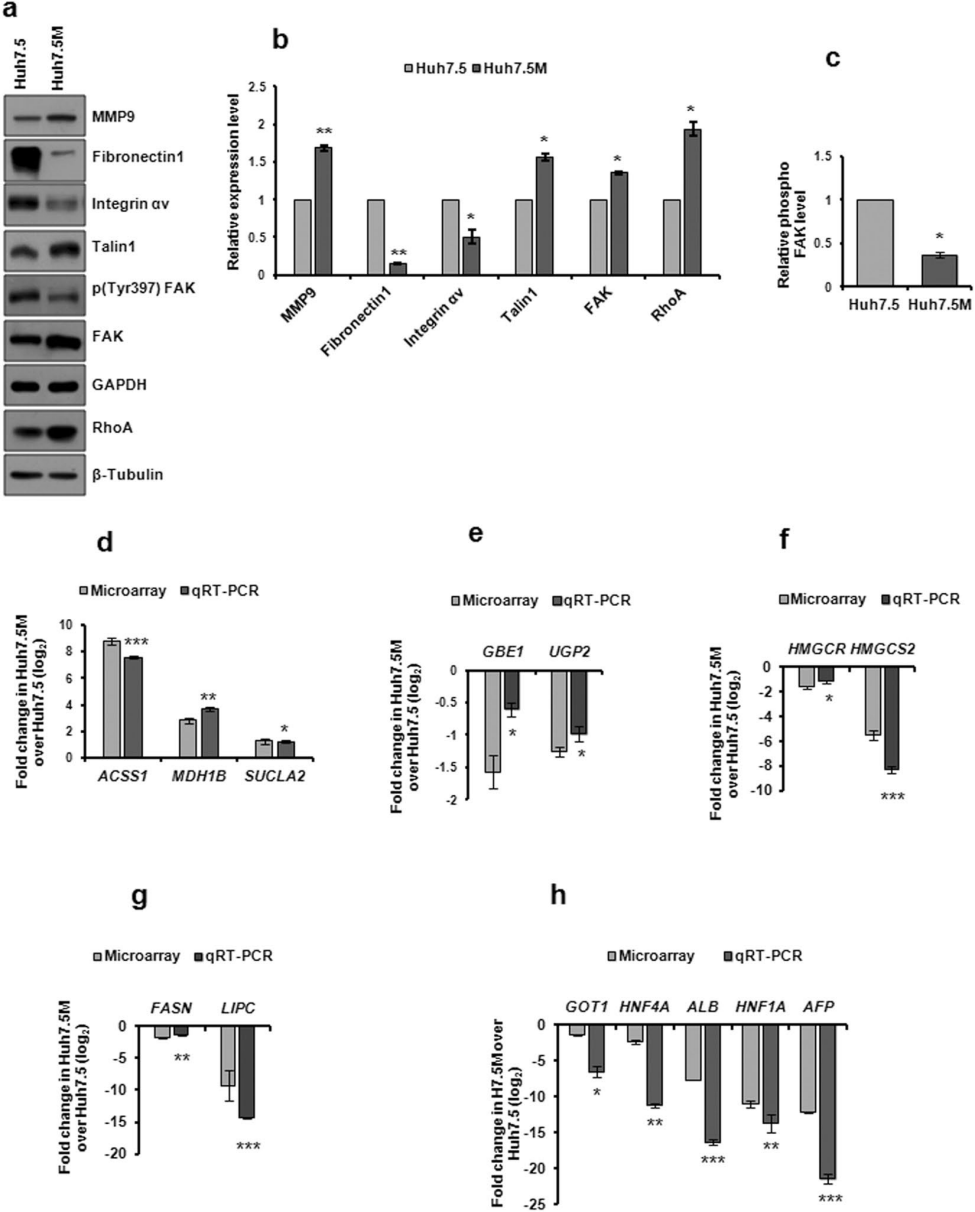
Validation of microarray results. (**a**) Huh7.5 and Huh7.5M cells were cultured up to 70% confluency, harvested and lysates prepared. Equal quantities of lysates were electrophoresed and subjected to immunobloting against the antigen mentioned. (**b**) Quantitative representation of results from (**a**). Intensities of the bands were analyzed by densitometry, normalized to those of the corresponding β-Tubulin or GAPDH and plotted graphically. (**c**) Comparison of FAK phosphorylation between the two cells. The intensities of the corresponding bands were normalized against that of the total protein and further normalized against those of GAPDH. Relative expressions of representative genes from TCA and OXPHOS (**d**), glycogen biosynthesis (**e**), cholesterol and bile acid biosynthesis (**f**) and fatty acid biosynthesis and degradation (**g**) pathways in Huh7.5 and Huh7.5M cells, quantified by qRT-PCR as described earlier. (**h**) demonstrates relative expression levels of representative liver-specific genes in Huh7.5 and Huh7.5M cells, quantified by qRT-PCR. *, ** and *** indicate *P*-values < 0.05, < 0.005 and < 0.0005 respectively.

Extracellular matrix (ECM) components and membrane receptors underwent extensive regulation of expression during EMT (Supplementary Table S3). Both cellular formats expressed a large number of these genes exclusively, suggesting a complex interplay. MMP9, a marker associated with EMT was elevated in Huh7.5M cells (Fig. 2a and b). The perplexing downregulation of *FN1* in Huh7.5M cells was further validated by immunobloting (Fig. 2a and b). *ITGA8* was substantially over-expressed in Huh7.5M cells (Supplementary Table S3), while Huh7.5 cells expressed increasing amounts of Integrin αv. Surprisingly, Y397 phosphorylation of focal adhesion kinase (FAK), the major downstream facilitator of integrin initiated signaling, was inhibited in Huh7.5M cells (Fig. 2a and c). Talin1, an integral member of integrin signaling, and RhoA, a key regulator of actin cytoskeleton and migration, were expressed at higher levels in Huh7.5M cells (Fig. 2a and b). Thus, ECM reorganization and integrin signaling appears anything but straightforward and careful analysis is required for identifying its crucial role in metastasis.

### EMT causes overhaul of metabolism

Cells undergo extensive metabolic reprogramming during tumorigenesis^37^. Otto Warburg reported in 1956 that cancer cells use glycolysis for their energy demand over oxidative phosphorylation (OXPHOS) under aerobic condition^38^. However, certain recent studies demonstrated that they can also utilize OXPHOS for their energy production^39, 40^. We sought to find out if epithelial and mesenchymal cancer cells differ in their choice of mechanism for energy production^41^. Major enzymes in glycolysis pathway did not indicate any specific directional change in regulation, but four key enzymes catalyzing irreversible steps in gluconeogenesis, viz. *PC*, *FBP 1* and 2 and *G6PC* were transcriptionally activated in Huh7.5 cells (Supplementary Table S4). Glycolysis feeds TCA cycle for the generation of energy by OXPHOS. Microarray analysis revealed that significant number of enzymes involved in TCA cycle and OXPHOS were overexpressed in Huh7.5M cells (Fig. 2d). These results indicate that Huh7.5M cells utilize OXPHOS for their energy demand as reported earlier^40^. In addition to energy production, these pathways also generate building blocks for generation of other macromolecules. Pentose phosphate pathway (PPP) that is associated with glycolytic pathway provides substrates for the generation of amino acids and nucleotides. Several enzymes participating in PPP (*H6PD*, *RGN*, *RPE* and *RBKS*) were transcriptionally repressed in Huh7.5M cells. Glucose is converted to glycogen in the liver and many enzymes engaged in glycogen biosynthesis pathway were transcriptionally underexpressed in Huh7.5M cells (Fig. 2e), indicating that these cells have lost certain properties characteristic to hepatocytes. Further substantiating this observation, key genes involved in cholesterol and bile acid biosynthesis pathway were depleted in Huh7.5M cells (Fig. 2f and Supplementary Table S4). Additionally, several genes encoding enzymes in the fatty acid biosynthesis and its degradation pathway were suppressed in Huh7.5M cells (Fig. 2g and Supplementary Table S4).

### Huh7.5M cells have lost liver features

Hepatocytes are critical to a wide variety of metabolic and physiological functions in liver homeostasis. We sought to learn if EMT causes changes to the intrinsic liver properties. Suppression of *ALT* (*GPT*), *AST* (*GOT*), *AFP* and *ALB*, major liver markers, was evident in Huh7.5M (Fig. 2h and Supplementary Table S9). Molecules involved with detoxification, another important function assigned to liver, such as ABC transporters are considerably underexpressed in Huh7.5M cells (Supplementary Table S9). Strikingly, a large number of members of liver enriched protein families such as ABC family, APO family, C1-C8, Cytochrome 450 family, Fibrinogen family, SERPINs, SLC family, were also depleted in Huh7.5M cells alongside *ALDO*, *AHSG, FTH1*, *FTL*, *ORM1* and 2, *PLG*, and *POX1*–3 (Supplementary Table S9). Additionally, liver specific hepatocyte nuclear factors *HNF* *1*, *3*, *4* and *6* (Fig. 2h and Supplementary Table S9) expressions were critically depleted in Huh7.5M. The liver specific nuclear receptor signaling LXR-RXR-RAR for lipid and cholesterol metabolism also seems to be very active in Huh7.5 as compared to Huh7.5M cells. This clearly implies that when cells undergo EMT, they not only lose their epithelial characteristics, but also their cell-specific functions.

### EMT in Huh7.5M is not regulated by TGFβ/BMP signal pathways

**Figure 3:**
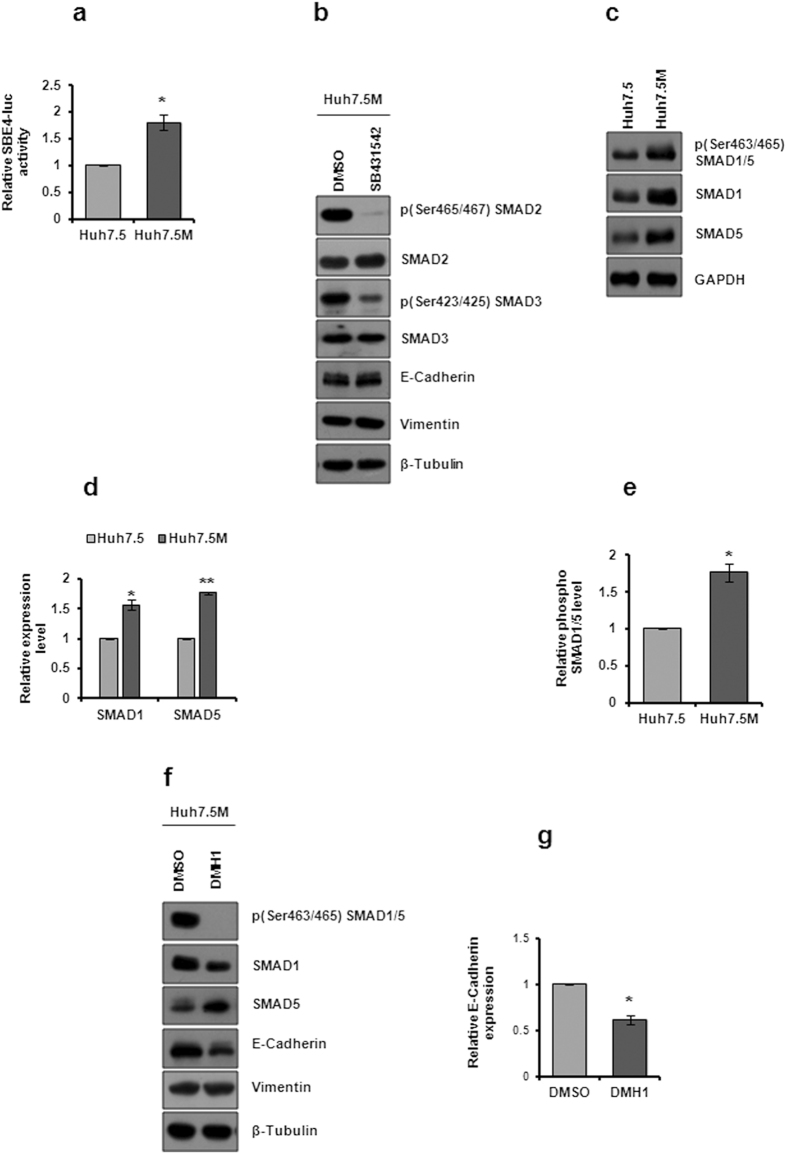
Analysis of TGFβ and BMP signaling in Huh7.5 and Huh7.5M cells. (**a**) Luciferase activity driven by SBE4 promoter in Huh7.5 and Huh7.5M cells. The cells were cultured and co-transfected with SBE4-Fluc and the control pCMV-Rluc plasmid and dual luciferase assay was performed as detailed in experimental procedures. Fluc/Rluc ratios were used for plotting the graph. (**b**) Effect of TGFβ inhibition on EMT. Huh7.5M cells treated with TGFβ receptor inhibitor SB431542 at 10 µM concentration for 1 hr and its effect on expression of EMT markers was analyzed by immunobloting. (**c**) Comparative analysis of BMP signaling in Huh7.5 and Huh7.5M cells by immunobloting. Cells cultured to 70% confluency were harvested and subjected to immunobloting against the corresponding molecules in BMP signaling. (**d**) Relative expression of SMAD 1 and 5 quantified by densitometry of the corresponding immunoblots from (**c**). Ratios from Huh7.5 cells were considered as 1 to which those of Huh7.5M cells were compared. (**e**) Quantitative representation of phosphorylation of SMAD 1 and 5 in Huh7.5 and Huh7.5M cells normalized to GAPDH. (**f**) Effect of BMP inhibition on EMT markers in Huh7.5M cells. Cells were treated with 2 µM DMH1 for 1 hr and its effects were analyzed by immunobloting. (**g**) Change in E-Cadherin expression upon BMP inhibition was represented graphically after densitometric analysis. * and ** indicate *P*-values < 0.05 and < 0.005 respectively.

We had previously demonstrated that TGFβ was unable to induce EMT in Huh7.5 cells^24^. Upon TGFβ treatment, E-Cadherin levels were augmented in Huh7.5 cells^24^. Interestingly, SMAD 2 and 3 phosphorylations remained elevated in Huh7.5 cells than the mesenchymal Huh7.5M cells. To gain further insight into the activation of TGFβ pathway, we performed SBE4 promoter activity by luciferase assay in Huh7.5 and Huh7.5M cells. Huh7.5M cells displayed considerably higher SBE4 activity than Huh7.5 cells suggesting that TGFβ pathway is probably more active in them (Fig. 3a). Further, inhibition of TGFβ signal pathway in Huh7.5M cells by pharmacological inhibitor SB431542 had no effect on the expression of either E-Cadherin or Vimentin (Fig. 3b), confirming the non-engagement of TGFβ pathway in EMT in Huh7.5M cells.

Unlike TGFβ pathway, BMP signaling was considerably active in Huh7.5M cells (Fig. 3c–e). SMAD1/5 phosphorylation was substantially induced in Huh7.5M cells (Fig. 3c and e) and was supported by higher expression (Fig. 3c and d). In agreement, *BMP* *1*, *5*, *6*, *7*, *8A* and *8B* expression were augmented in Huh7.5M cells (Supplementary Table S5). Contrary to expectation, inhibition of BMP pathway in Huh7.5M cells by DMH1 further suppressed E-Cadherin without altering Vimentin levels (Fig. 3f and g), indicating a confounding association of BMP pathway with EMT in these mesenchymal cells.

### NF-κB pathway and EMT

**Figure 4:**
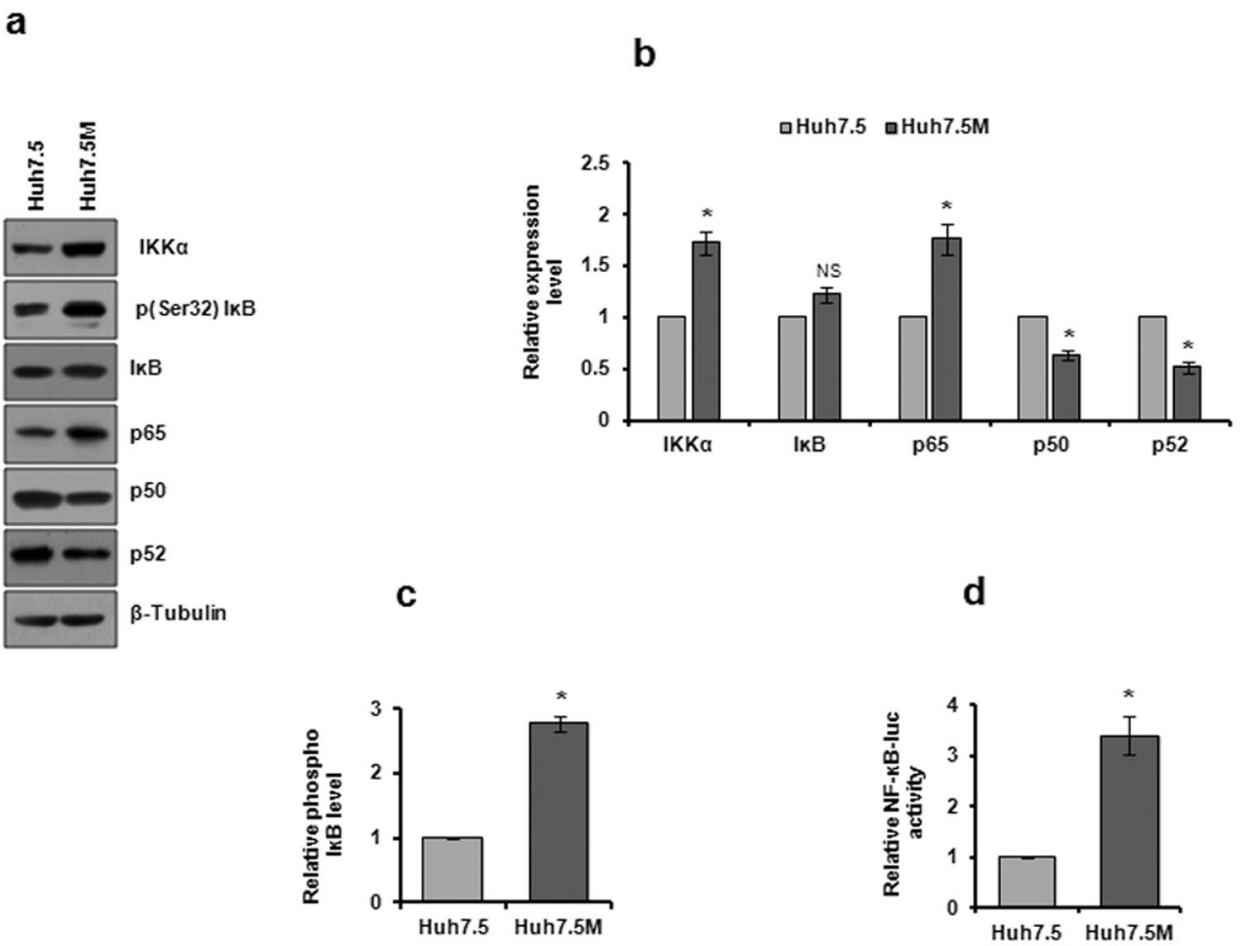
Analysis of NF-κB signaling in Huh7.5:Huh7.5M cells. (**a**) Immunobloting of major molecules in NF-κB pathway in cells cultured to 70% confluency. (**b**) Graphical representation of the expression levels of molecules through densitometry analysis of band intensities from (**a**). (**c**) Analysis of IκB phosphorylation in the two cells by densitometry from (**a**). (**d**) NF-κB responsive element reporter assay. Cells co-transfected with NF-κB-Fluc and pCMV-Rluc plasmids were harvested 30 hrs later and dual luciferase assay was performed. Relative Fluc/Rluc ratio was plotted in the graph. * indicates *P*-value < 0.05. NS-non significant.

Chronic inflammation activated by malignant cells sets a tumor conducive platform promoting carcinogenesis^42^. NF-κB, one of the most extensively studied molecules in inflammation, is reported to act as a tumor suppressor in normal cells, while as an oncogene in cancer cells^43^. Transcription profiling identified regulation of molecules participating in NF-κB pathway (Supplementary Table S6). Immunoblot profiling of Huh7.5 and Huh7.5M cells revealed interesting regulation of NF-κB pathway. Elevated p65 expression and increased IκB phosphorylation in Huh7.5M indicated activated NF-κB signaling in them (Fig. 4a–c). In tandem, p50 and p52 levels were restricted in Huh7.5M cells that could further induce NF-κB activity^44^. NF-κB luciferase reporter assays substantiated the active NF-κB signaling in Huh7.5M as compared to Huh7.5 cells (Fig. 4d).

### Unconventional GSK-3β/β-Catenin pathway maintains EMT in Huh7.5M cells

**Figure 5:**
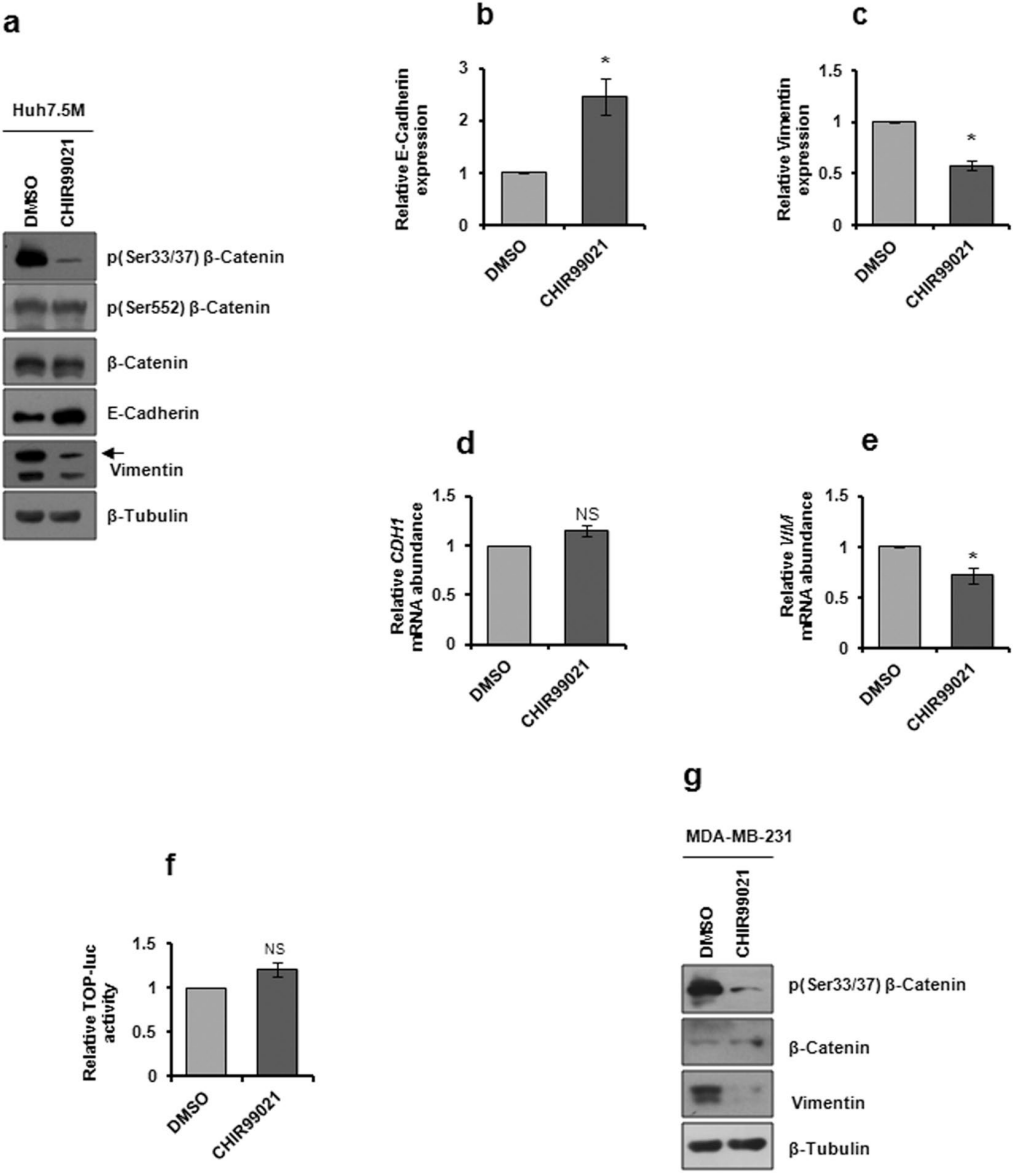
Analysis of GSK-3β signaling in Huh7.5M cells. (**a**) Huh7.5M cells were treated with 10 µM CHIR99021 for 1 hr at 70% confluency. Cells were harvested post-treatment and subjected to immunobloting. (**b**) and (**c**) describe quantitative representation of relative expressions of E-Cadherin and Vimentin in Huh7.5M cells from (**a**). (**d**) and (**e**) depict relative transcript levels of *CDH1* and *VIM* in Huh7.5M cells upon inhibition by CHIR99021 quantified by qRT-PCR. (**f**) Relative TOP-luciferase activity measured from Huh7.5M cells treated with CHIR99021. Dual luciferase assay was performed 30 hrs post-transfection of cells with TOP-Fluc and pCMV-Rluc plasmids. One hour prior to the harvest, cells were treated with the inhibitor. (**g**) GSK-3β inhibition and analysis of EMT markers in MDA-MB-231 cells similar to (**a**). 50 µM CHIR99021 was used in inhibition. * indicates *P*-value < 0.05. NS-non significant.

We previously reported that β-Catenin may not be a critical regulator of this EMT based on the absence of a credible change in β-Catenin levels between the two cells^24^. Contradicting this hypothesis, IPA of the transcription profiles flagged a highly active Wnt/β-Catenin signaling in Huh7.5M cells based on the upregulation of majority of its receptors and ligands (Fig. 1a and Supplementary Fig. S1; Table 1). Intriguingly, *APC*2, *AXIN 1* and *2* were also highly expressed in Huh7.5M cells (Supplementary Table S7). To verify the necessity of this pathway in EMT in Huh7.5M cells, we pharmacologically inhibited GSK-3β, a negative regulator of Wnt/β-Catenin by CHIR99021. GSK-3β inhibition led to considerable dephosphorylation of β-Catenin at S33/37 residues (Fig. 5a) without affecting S552 phosphorylation and total quantity. This indicated that GSK-3β inhibition possibly did not induce the stability and nuclear translocation of β-Catenin. In support, β-Catenin mediated TCF/LEF promoter activity was unaffected during the inhibition as revealed by TOP-FLASH reporter assay (Fig. 5f). Further, β-Catenin over-expression in Huh7.5 cells did nothing to promote EMT that was also consistent in another non-metastatic cell line, MCF-7 (Supplementary Fig. S2). Surprisingly, the inhibition led to augmented levels of E-Cadherin with concomitant suppression of Vimentin (Fig. 5a–c), both of which are indicators of MET induction. This result is in total contradiction to the current understanding of GSK-3β mediated regulation of β-Catenin where GSK-3β inhibition by Wnt ligands activates EMT through suppression of E-Cadherin. Suppression of Vimentin by CHIR99021 was consequent of its transcriptional inhibition while *CDH1* mRNA levels were unaffected by it (Fig. 5d and e). To further examine whether GSK-3β inhibition enhanced the stability of *CDH1* mRNA that can in turn increase E-Cadherin synthesis, full-length 3′UTR of *CDH1* mRNA was cloned downstream of Rluc in psiCHECK2 vector and luciferase activity was measured during GSK-3β inhibition in Huh7.5M cells. No change in relative Rluc activity was detected during the inhibition (Supplementary Fig. S3), suggesting that increased E-Cadherin levels is not the result of post-transcriptional events, but could be an outcome of stabilization of protein. GSK-3β inhibition had little effect on EMT marker levels in Huh7.5 cells (Supplementary Fig. S4). Interestingly, GSK-3β inhibition in another highly metastatic and non-hepatic cell line MDA-MB-231caused Vimentin depletion without promoting E-Cadherin expression (Fig. 5g). Therefore, Vimentin appears to be a general target during GSK-3β inhibition, implying that GSK-3β participates in the protection of mesenchymal characteristics in tumor cells. These results demonstrate a novel mode of action of GSK-3β that is probably critical in the maintenance of mesenchymal properties in Huh7.5M cells. Detailed studies are necessary to identify the significance of Wnt/β-Catenin in this scheme of regulation.

### p38MAPK and ERK1/2 regulate EMT through GSK-3β

**Figure 6:**
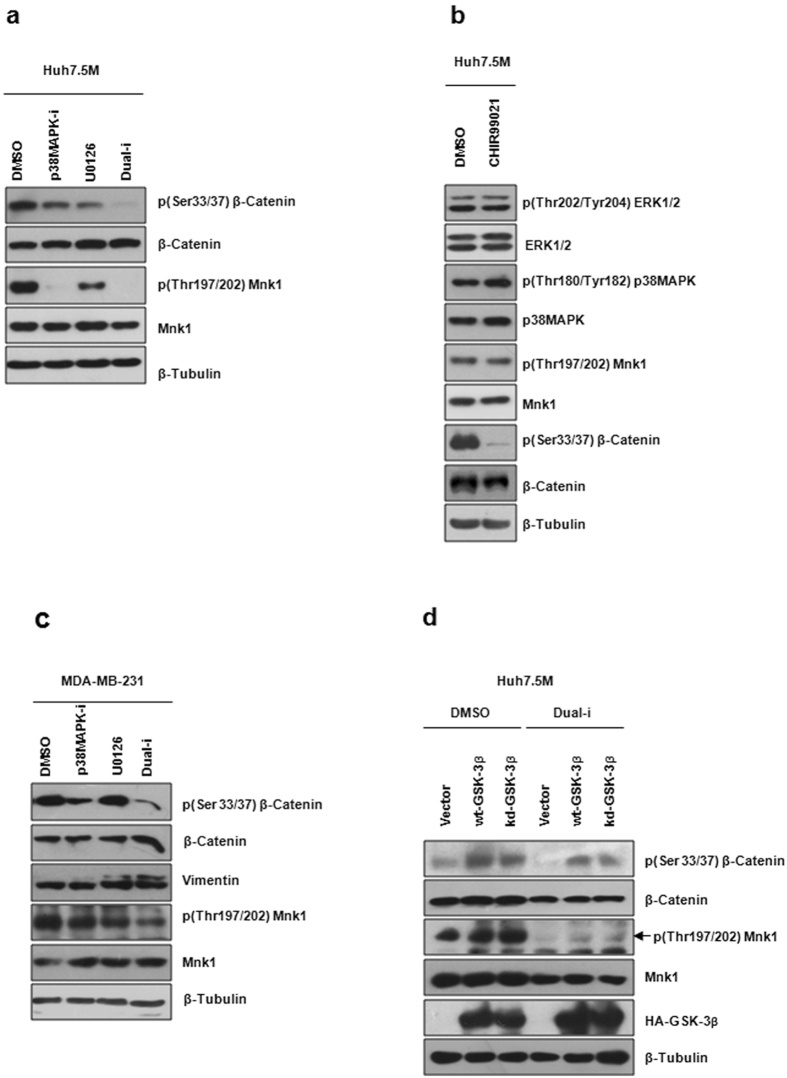
Regulation of GSK-3β signaling by p38MAPK and ERK1/2 in Huh7.5M cells. (**a**) Huh7.5M cells treated with 50 µM of p38MAPK inhibitor VIII or 100 µM of U0126 or both of them for 1 hr were lysed and the effects were analyzed by immunobloting. (**b**) Effect of GSK-3β inhibition on p38MAPK and ERK1/2 in Huh7.5M cells treated with 10 µM CHIR99021 for 1 hr analyzed by immunobloting as in (**a**). (**c**) Consequence of p38MAPK and ERK1/2 inhibitions on GSK-3β signaling in MDA-MB-231 cells. Cells were treated with 50 µM of p38MAPK inhibitor VIII or 50 µM of U0126 individually or together for 1 hr before harvesting. (**d**) Effect of wt and kd-GSK-3β expressions on β-Catenin phosphorylation during p38MAPK and ERK1/2 dual inhibition in Huh7.5M cells. Cells were transfected with the respective plasmid for 48 hrs before the inhibitions.

We had previously demonstrated that p38MAPK and ERK1/2 independently control EMT in Huh7.5M cells as their inhibitions induced MET^24^. Since GSK-3β inhibition also resulted in MET, we asked if the two MAPK pathways network with GSK-3β in the process. Huh7.5M cells were inhibited with p38MAPK VIII inhibitor or with U0126 or in combination for 1 hr. Mnk1 dephosphorylation confirmed the inhibition of the target molecules (Fig. 6a). Interestingly, GSK-3β was inhibited independently by p38MAPK and MEK inhibitors and more potently during dual inhibition, suggesting that GSK-3β was downstream to the MAPKs (Fig. 6a). In agreement, neither of the MAPKs was inhibited during GSK-3β inhibition (Fig. 6b). To further verify if this regulation is universal, MDA-MB-231 cells were treated with inhibitors as above. Unlike in Huh7.5M cells, only p38MAPK inhibition resulted in strong inhibition of β-Catenin phosphorylation suggesting the inhibition of GSK-3β activity (Fig. 6c). However, this inhibition did not influence EMT marker levels. Thus, considerable variation exists in this regulatory network among various cell lines.

To further validate these findings, we treated Huh7.5M cells ectopically expressing wt or kinase dead (kd) mutant forms of GSK-3β (K85A) with MAPK dual inhibitors. Over-expression of wt- GSK-3β induced β-Catenin phosphorylation (Fig. 6d). Unexpectedly, kd-GSK-3β also induced an equivalent β-Catenin phosphorylation, suggesting that the active site is dispensable for GSK-3β mediated β-Catenin phosphorylation. Mnk1 was effectively dephosphorylated by the inhibitors, confirming the inhibition of the two MAPKs (Fig. 6d). Dual inhibition caused inhibition of β-Catenin phosphorylation (Fig. 6d; lanes 5 and 6) in comparison with DMSO controls (Fig. 6d; lanes 2 and 3), as seen earlier. No perceivable changes in β-Catenin phosphorylation were observed between the wt- and the kd-GSK-3β expressing cells upon inhibitor treatment. However, the response of β-Catenin phosphorylation to expression of GSK-3β and to dual inhibition confirms that GSK-3β is regulated by the two MAPKs. Interestingly, GSK-3β expression caused no change in EMT markers in Huh7.5 and MCF-7 cells (Supplementary Fig. S5), highlighting the cell-specificity of signaling networks. Thus, we identified that p38MAPK and ERK1/2 maintain EMT in Huh7.5M cells through a novel mechanism involving GSK-3β.

## Discussion

Information generated over the past decade reiterates that cancer EMT is a vast and diverse process with significant variation observed in the induction mechanism and the extent of EMT. During EMT, the cell undergoes dramatic changes. On either end of the transcriptional reprogramming, the cell experiences changes at signaling and metabolic levels. To understand the EMT process in a holistic way one should be able to connect all these major changes to one another. Despite the complexity in the presentation of EMT, roles of the canonical EMT-TFs have been undoubtedly proven. Intriguingly, Snail and Zeb1 were detected at robust levels in the epithelial Huh7.5 and their over-expression had no change on the cells^24^, indicating that additional factors were required to promote EMT. At this outset, we profiled transcriptomes of Huh7.5 and Huh7.5M cells and also studied the major pathways that drive EMT. Our analysis identified a large-scale transcriptional reprogramming and several factors with potential to influence EMT. However, the novel factors regulating this atypical EMT remain elusive.

It is crucial to note that only a little over a third of the EMT core gene list underwent differential expression as predicted in Huh7.5:Huh7.5M system. This is a major deviation from the canonical EMT process and straightaway challenges the significance of over half the genes in the EMT core genes list. Since many important mesenchymal markers such as Fibronectin, Snail, Zeb1, N-Cadherin and β-Catenin were detected in Huh7.5 cells either at higher or at similar levels to Huh7.5M cells^24^, Huh7.5 cells have presumably crossed the initiation stages of EMT and therefore are not purely epithelial based on EMT scoring. However, they retain the typical morphological and phenotypic characteristics of epithelial cells. Therefore, EMT as a process has tremendous in-built flexibility to hold cells in any of the multiple stages in a broad spectrum where cells in each of the stages could be substantially varying from each other in terms of its properties and capabilities. In the same measure, our studies suggest that E-Cadherin is one of the last markers discarded during EMT and thus could be misleading to be used as the sole marker. Thus, our studies have identified two cell types placed at two distinct stages of EMT, but varying phenomenally in their phenotypes. Capturing such stages would be extremely challenging when EMT is attempted in cultures using the standard inducers.

Physiological EMT is a major process during embryogenesis. Embryogenesis and cancer development share many mechanisms^45^. Several transcription factors crucial for vertebrate development were regulated during EMT in Huh7.5M (Supplementary Table S8). Among them, *GSC*, *LHX*, *DLX*, *EYA* and *GLI* family were upregulated in Huh7.5M. Many novel factors that have previously been associated with embryo development but not with cancer, such as *ATOH*, *TSHZ*, *BNC2* and *OLIG*, were identified in this study (Supplementary Table S8). Our studies stress on the necessity for examining the common mechanisms shared by the two processes to gain deeper insights into cancer metastasis. The mechanism of operation of such “Onco – Developmental TFs” in tumor progression could be an exciting field to explore in detail.

Inhibition of GSK-3β was anticipated to activate EMT in Huh7.5 cells. However, expression of Wnt antagonists *DKK*, *WIF1* and *SFRP* in these cells (Supplementary Fig. S1) could have jeopardized the receptor signaling. Nevertheless, the indifference of Huh7.5 to GSK-3β inhibition and the strikingly contrasting results in Huh7.5M immediately question the conventional standing in the field. β-Catenin stability was not visibly enhanced by GSK-3β inhibition in Huh7.5M cells since its levels remained unaltered. Additionally, steady state phosphorylation of β-Catenin at S552 coupled with unaltered TCF/LEF promoter activity suggests that nuclear translocation of β-Catenin was not activated by GSK-3β inhibition. Therefore, increasing E-Cadherin levels mediated through GSK-3β inhibition was not enforced through transcriptional, but through post-transcriptional or post-translational mechanisms. Enhanced stabilization of E-Cadherin appears to be a strong possibility in the absence of involvement of 3′UTR mediated regulation. The stabilization of E-Cadherin by β-Catenin dynamics is well established. In the absence of β-Catenin nuclear activation, *VIM* transcriptional repression appears to be β-Catenin independent. Since GSK-3β inhibition induced MET, this system could be used in understanding the details of the mechanism. This result was partly reproduced in MDA-MB-231 cells, suggesting that GSK-3β could be either a promoter or stabilizer of mesenchymal properties. This role of GSK-3β is at direct odds with its established identity as a pro-apoptotic molecule and thus could be a potential drug target in cancer metastasis. To the best of our knowledge, no such role of GSK-3β has been hitherto reported. Our results demonstrate a very unconventional GSK-3/β-Catenin signaling and mandate a careful approach while targeting Wnt pathway for cancer therapy. This system also offers an alternate platform for studying the promiscuous signaling by Wnt/GSK-3/β-Catenin pathway.

GSK-3β inhibition and over-expression made no impact on EMT in Huh7.5 cells. MCF-7 cells also were non-responsive to GSK-3β expression. This was in stark contrast to Huh7.5M and MDA-MB-231 cells both of which were sensitive to GSK-3β mediated EMT regulation. Our studies underline the requirement of additional factors for GSK-3β to perform this function. Therefore, the system that we characterized could be a potential tool in identifying these factors and unraveling the mechanism through which GSK-3β regulates EMT.

**Figure 7:**
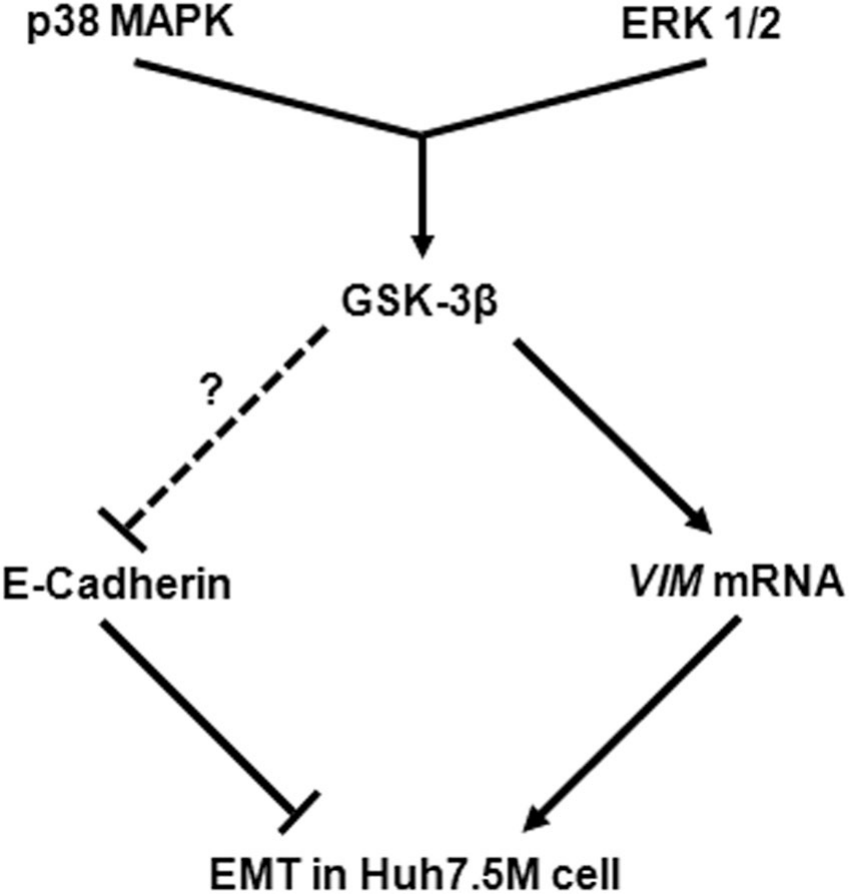
Model for the regulation of mesenchymalness in Huh7.5M cells. p38MAPK and ERK1/2, independently and together, activate GSK-3β. Inhibition of either of these MAPKs in Huh7.5M cells resulted in dephosphorylation of β-Catenin at S33/37, a target of GSK-3β. Separately, GSK-3β inhibition augmented the levels of E-Cadherin without a transcriptional regulation. In the absence of 3′UTR mediated mRNA regulation, enhanced stability provided by dephosphorylated cytoplasmic β-Catenin appears to be the mechanism behind the augmented E-Cadherin levels. On the other hand, GSK-3β inhibition transcriptionally suppressed *VIM*, thus inducing MET. In the absence of an apparent change in the nuclear activity of β-Catenin, transcriptional regulation of *VIM* appears β-Catenin independent. Through this model, we demonstrate a novel regulation of EMT wherein p38MAPK and ERK1/2 maintain mesenchymalness in Huh7.5M cells through GSK-3β mediation.

p38MAPK and ERK1/2 was found to be crucial in helping Huh7.5M cells maintain the mesenchymalness and this was achieved through GSK-3β. To the best of our knowledge, this is the first report demonstrating the pro-EMT role of GSK-3β and its activation by the MAPKs. Previous reports demonstrate the negative regulation of ERK1/2 by GSK-3β^46^. Antagonistic effect of ERK1/2 on GSK-3β mediated apoptosis is also reported^47^. p38MAPK is also shown to inactivate GSK-3β by phosphorylation of its C-terminal residues^48^. However, our report is novel, as GSK-3β works in tandem with the MAPKs in Huh7.5M (Fig. 7). Notwithstanding the induction of MET during GSK-3β inhibition in MDA-MB-231 cells, MAPK inhibitions could not reproduce these results, suggesting the involvement of additional factors. Further investigation is required to understand how the MAPKs activate GSK-3β in Huh7.5M cells in the context of active Wnt/β-Catenin signaling.

In agreement with our previous report, TGFβ inhibition did not cause any change in EMT status in Huh7.5M cells. Increased SMAD2/3 phosphorylation was more evident in Huh7.5 cells as compared with Huh7.5M^24^. Albeit, TGFβ pathway was more active in Huh7.5M cells as depicted by the enhanced SBE4 activity. The implications of TGFβ in Huh7.5:Huh7.5M system is ambiguous. Notwithstanding the activation of BMP signaling in Huh7.5M cells, its inhibition caused little change in Vimentin expression. However, it is quite possible that the pathway modulates activities that are accessory to EMT. Which signaling pathways would have contributed meaningfully to EMT? What could be the contributions of BMP/NF-κB pathways even though Wnt seems to be the most influential? Do they collaborate with Wnt pathway?

A striking consequence to EMT was the loss of several liver specific molecules with key functions. Despite being tumorous, Huh7.5 cells retained key “hepato-features” that were lost in Huh7.5M cells. Liver being a major organ in human physiology, the loss of liver functions as a consequence to EMT would severely cripple the metabolic functioning of the patient who is already living a compromised life. Liver also converts several pro-drugs to active drugs and hence proper metabolic functioning of liver is critical to the efficacy of treatment itself. To the best of our knowledge, this is the first study demonstrating large-scale loss of liver properties following EMT.

What is atypical about Huh7.5:Huh7.5M system and what do we learn from it? To the best of our understanding a few points justify our claim of it being atypical. Firstly, expression of EMT-TFs Snail and Zeb in Huh7.5 cells with concomitant expression of epithelial markers is strange. Secondly, detection of other mesenchymal markers such as N-Cadherin, Fibronectin and β-Catenin co-residing with epithelial markers is unique. The dispensability of these factors in the perceivably epithelial cells needs further interrogation. Thirdly, MAPK-GSK-3β signaling behaved in total contradiction to its conventional role in EMT. Even with activation of Wnt and BMP pathways in Huh7.5M, their regulation was anything but straightforward. Since such mechanisms do exist in cultured cells, their existence *in vivo* cannot be ruled out. So, these results not only highlight the dynamic and diverse nature of mechanisms that drive EMT in different biological systems including numerous cancer EMT models, but underline the necessity of scrutiny that should be exercised while extrapolating results from them in the search for therapeutic targets.

## Methods

### Cell culture and reagents

Huh7.5 and Huh7.5M cells were cultured as described earlier^24^. MCF-7 and MDA-MB-231 cells were cultured in DMEM with 10% FBS. GSK-3β inhibitor CHIR99021, TGFβ inhibitor SB431542 and BMP inhibitor DMH1 were from Sigma. U0126 and p38MAPK inhibitor VIII were from Merck Millipore. All the primary antibodies were from Cell Signaling Technology (Danvers, MA) except for Integrin αv and IκB (Santa Cruz Biotechnology, Dallas, TX); FAK (Merck Millipore); MMP9 (Bioss Antibodies, Woburn, MA); Y397 FAK, GAPDH and β-Tubulin (Thermo Fisher Scientific, Waltham, MA). Secondary antibodies were from Jackson ImmunoResearch, West Grove, PA. HA-tagged human β-Catenin and GSK-3β coding sequences were cloned in pcDNA4/TO plasmid. kd-GSK-3β (K85A) was generated by site directed mutagenesis. All the clones and mutant were and confirmed by Sanger’s sequencing. Sequences of the oligos used are provided in Supplementary Table S11.

### Whole transcriptome profiling

Huh7.5 and Huh7.5M cells were grown to reach 70–80% confluency. Cells were washed twice with ice cold 1X PBS. Cells were harvested using 0.05% Trypsin-EDTA and washed with 1X PBS. Total RNA was prepared using MN nucleospin RNA kit and used for microarray analysis. Samples were prepared from biological replicates belonging to two separate passage numbers. RNA samples were subjected to microarray analysis as detailed below.

### Microarray analysis

Sample preparation, processing and analysis of the results were performed by Genotypic Technologies, Bangalore. The samples that passed integrity analysis by Bioanalyzer (Agilent Technologies, Santa Clara, CA) were labeled using Agilent Quick Amp Kit. 500 ng of total RNA was reverse transcribed using oligo-dT primer tagged to T7 promoter sequence. cDNA thus obtained was converted to double stranded cDNA in the same reaction. Further, the cDNA was converted to cRNA by *in vitro* transcription using T7 RNA polymerase. During cRNA synthesis Cy3 dye was incorporated into the newly synthesized strands. cRNA obtained was cleaned up using RNeasy columns (Qiagen, Venlo). 600 ng of labeled cRNA were hybridized on the array using the Gene Expression Hybridization kit (Agilent). Hybridized slides were washed and were subsequently scanned on a G2505C scanner (Agilent).

### Microarray Data Analysis

Images were quantified using Feature Extraction Software (Version-10.7, Agilent). Feature extracted raw data was analyzed using GeneSpring GX Version 12.0 from Agilent. Normalization of the data was done in GeneSpring GX using the 75th percentile shift and normalized to specific control samples. Genes with average fold change ≥(log_2_ 1) and ≤(log_2_ (−1)) in Huh7.5M cells over those in Huh7.5 were considered upregulated and downregulated respectively. Differential gene expression profile from biological replicates was generated based on the above criteria. Many genes that showed significant change in the replicates, but failed to meet stringent statistical significance were also considered for comparison. This is based on our conviction that genes that undergo consistent differential expression would have biological significance and hence should be considered significant, irrespective of their statistical significance. Genes were classified using GeneSpring GX and Biointerpreter-Biological Analysis softwares (Genotypic Technology). Ingenuity Pathway Analysis (IPA, Qiagen) was used to study differentially regulated pathways. The data can be accessed at GEO accession number GSE78085.

### Quantitative real time RT-PCR

Total RNA was isolated as mentioned above. cDNA was generated using Primescript reverse transcriptase (Takara Bio, Mountain View, CA). 150 ng of cDNA samples were amplified using gene specific primers and SYBR Premix Ex Taq (Takara Bio) in Lightcycler 480 (Roche Molecular Diagnostics, Pleasanton, CA). Fold changes between the samples were calculated using ΔΔ Cp values with GAPDH serving as internal control. Average fold changes from a minimum of three independent experiments were graphically represented. Primer sequences used in the reactions are listed in Supplementary Table S10. Oligos for *CDH1*, *VIM* and *GAPDH* quantification were described previously^24^.

### Immunobloting and analysis

Cultured cells were harvested, washed and lysed with NP-40 lysis buffer^24^. Equal quantities of proteins from lysates were electrophoretically resolved in SDS-PAGE, transferred onto 0.45 µ PVDF membrane and immunobloted as described elsewhere^24^. Densitometric analyses of the bands were performed using ImageJ software.

### Luciferase assays

Cells were grown to 70% confluency at which point they were transfected with luciferase vectors using Lipofectamine 2000 (Thermo Fisher Scientific). Cells were harvested at 30 hrs post-transfection and luciferase assay was performed using dual luciferase reagent (Promega, Fitchburg, WI) according to the manufacturer’s protocol. M50 Super 8x TOP Flash was a gift from Randall Moon^49^ (Addgene #12456). NF-κB-luciferase reporter plasmid was gifted by Dr. Ghanshyam Swarup^50^. SBE4-luc was gift from Bert Vogelstein^51^ (Addgene plasmid # 16495). pCMV-Rluc plasmid with Rluc under CMV promoter was used for normalization^52^.

### Statistical significance

Experimental results are represented as Mean ± SEM of a minimum three independent experiments. For *P*-value calculation, paired-end, two-tailed, t-test was performed. *, ** and *** indicate *P*-values < 0.05, < 0.005 and < 0.0005 respectively.

## Electronic supplementary material

Supplementary Information 
