# Supplementary material for: Unconventional MAPK-GSK-3β Pathway Behind Atypical Epithelial-Mesenchymal Transition In Hepatocellular Carcinoma
Source: Sci Rep. 2017 Aug 18;7:8842. doi: 10.1038/s41598-017-09179-0 (PMC5562823; doi:10.1038/s41598-017-09179-0)
Supplement: Supplementary file 1 — Supplementary Information [file 41598_2017_9179_MOESM1_ESM.pdf]

Unconventional MAPK-GSK-3β Pathway Behind Atypical Epithelial-Mesenchymal Transition In Hepatocellular Carcinoma

Sana Parveen[a], Dhiviya Vedagiri[a], Hitha Gopalan Nair[a], Haripriya Parthasarathy[a] and Krishnan Harinivas Harshan[a]*

[a]CSIR-Centre for Cellular and Molecular Biology, Hyderabad- 500007, India

*To whom correspondence should be addressed: Dr. Krishnan Harinivas Harshan. Tel.: +91 4027192925, email: hkrishnan@ccmb.res.in

S. No.	Figures/Tables	Page No.
1	Supplementary Figure S1	3
2	Supplementary Figure S2	4
3	Supplementary Figure S3	5
4	Supplementary Figure S4	6
5	Supplementary Figure S5	7
6	Supplementary Table S1	8
7	Supplementary Table S2	11
8	Supplementary Table S3	16
9	Supplementary Table S4	19
10	Supplementary Table S5	23
11	Supplementary Table S6	24
12	Supplementary Table S7	26
13	Supplementary Table S8	28
14	Supplementary Table S9	29
15	Supplementary Table S10	35
16	Supplementary Table S11	37

Figure S1

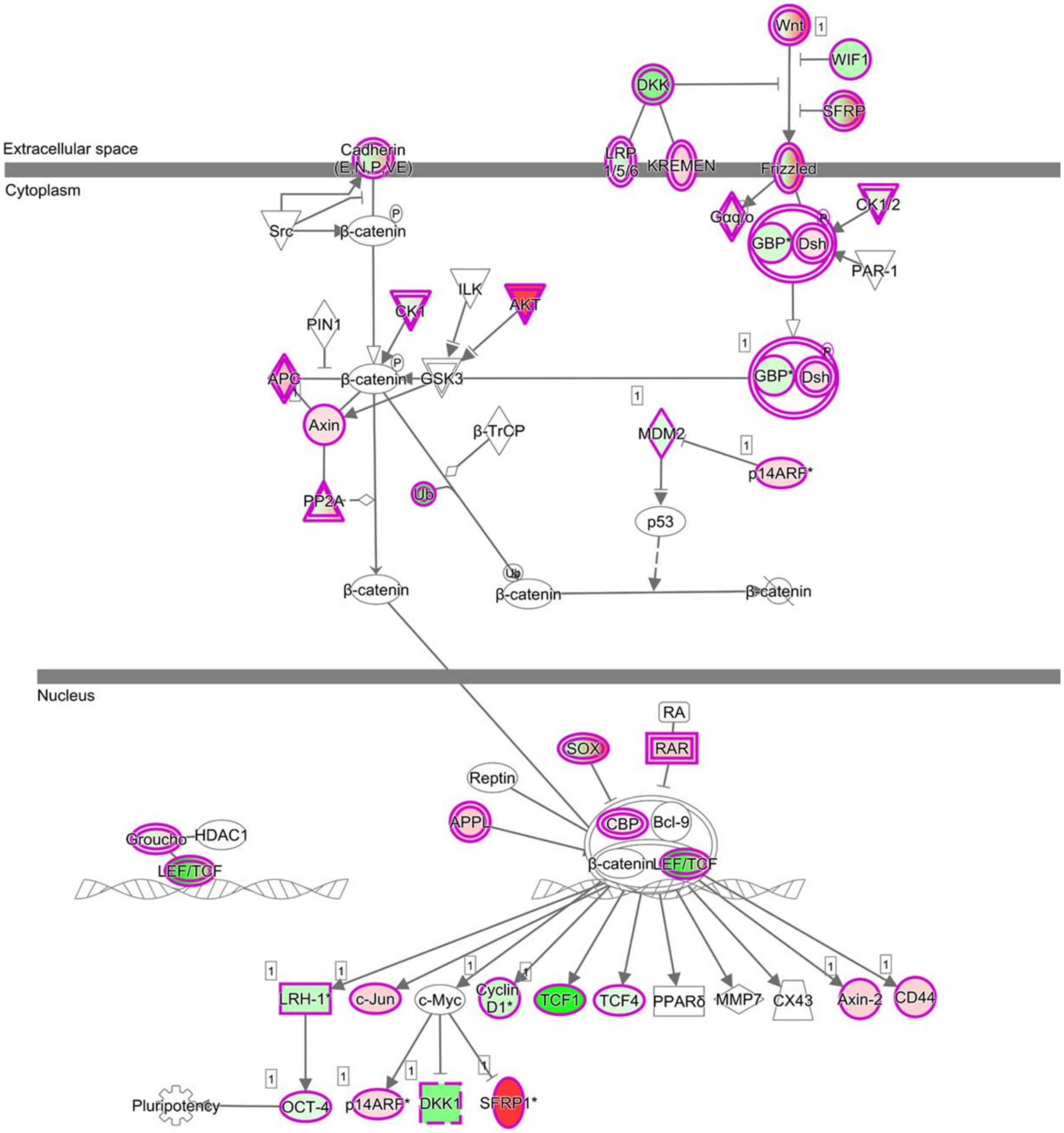

Figure S1. Regulation of Wnt/β-Catenin signaling in Huh7.5:Huh7.5M cell system identified by IPA. Green and red shades indicate downregulation and upregulation in Huh7.5M cells, respectively.

Figure S2

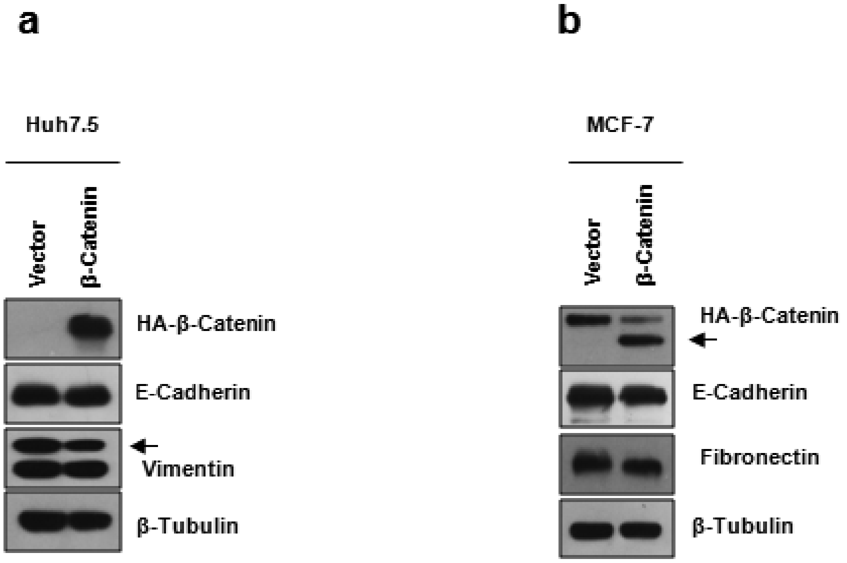

Figure S2. Ectopic expression of β-Catenin and its effect on EMT in (a) Huh7.5 and (b) MCF-7 cells. Cells transfected with β-Catenin over-expressing plasmids or the control empty vectors were analyzed for EMT marker expression by immunobloting. Fibronectin was detected in MCF-7 cells as Vimentin was undetectable.

Figure S3

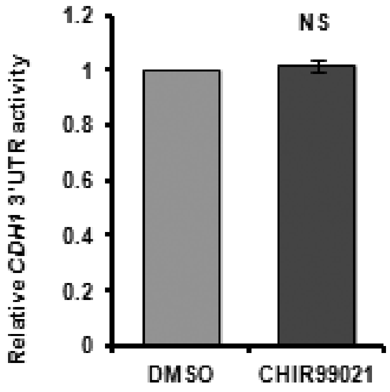

Figure S3. Effect of GSK-3β inhibition on *CDH1* 3'UTR activity. Huh7.5M cells were transfected with *CDH1* 3'UTR in psiCHECK2 vector. Cells were treated with 10μM CHIR99021 for an hour before harvesting. Luciferase activity was measured 30hrs post transfection

Figure S4

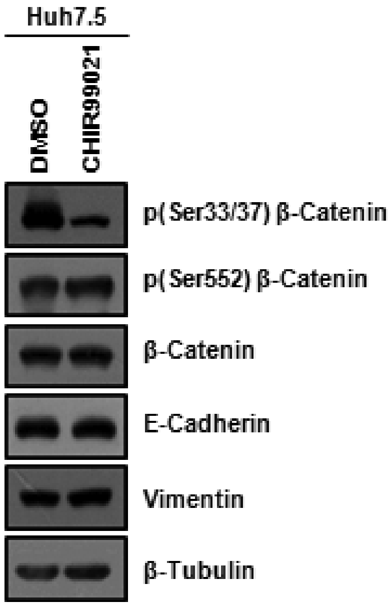

Figure. S4. Effect of GSK-3β inhibition on EMT in Huh7.5 cells. Cells were treated with 10μM CHIR99021, harvested at 1 hr post-treatment and subjected to immunobloting.

Figure S5

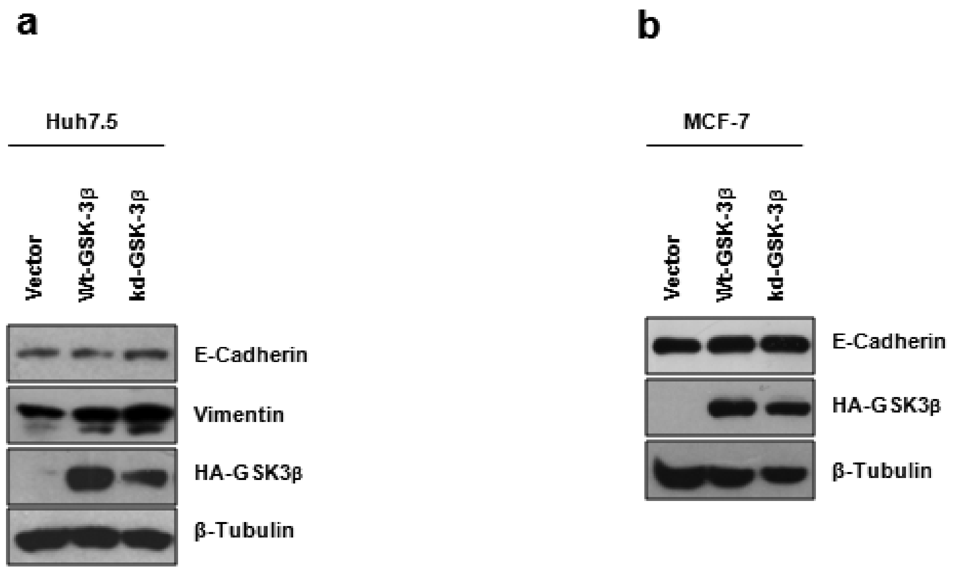

Figure S5. Consequence of over-expression of GSK-3β in (a) Huh7.5, and (b) MCF-7 cells. HA-tagged wt- or kd- mutant of GSK-3β variant expressing plasmid was transfected into the cells and its effect on EMT was analyzed by immunobloting.

Supplementary Table S1- Analysis of EMT core gene expression

Up and Down indicate genes overexpressed and underexpressed in Huh7.5M cells respectively.

Fold change values ≥1 and ≤-1 indicate genes upregulated and downregulated in Huh7.5M cells respectively.

Gene Symbol	Accession Number	Fold change (log_2) Set1	Fold change (log_2) Set2	Average Fold change (log_2)	*P* -value	Groger's Prediction	In this study
CDH11	NM_001797	5.66	6.30	5.98	0.034	Up	Up
CDKN2C	NM_001262	1.66	1.87	1.76	0.038	Up	Up
COL1A1	NM_000088	5.59	5.55	5.57	0.002	Up	Up
COL3A1	NM_000090	1.89	4.08	2.98	0.224	Up	Up
COL5A1	NM_000093	3.43	3.41	3.42	0.002	Up	Up
COL6A1	NM_001848	5.98	5.27	5.62	0.040	Up	Up
CYP1B1	NM_000104	0.83	2.56	1.70	0.300	Up	Up
DCN	NM_001920	2.05	1.55	1.80	0.088	Up	Up
EML1	NM_001008707	2.03	1.73	1.88	0.051	Up	Up
EMP3	NM_001425	4.78	4.26	4.52	0.037	Up	Up
FGF2	NM_002006	0.98	1.67	1.32	0.162	Up	Up
FSTL1	NM_007085	2.48	3.35	2.92	0.094	Up	Up
LTBP1	NM_206943	3.30	2.78	3.04	0.054	Up	Up
MAP1B	AK055112	0.82	1.30	1.06	0.142	Up	Up
MMP2	NM_004530	2.66	3.42	3.04	0.079	Up	Up
NID2	NM_007361	3.01	4.45	3.73	0.121	Up	Up
PLAT	NM_000930	9.14	7.23	8.19	0.074	Up	Up
PMP22	NM_000304	2.55	4.70	3.63	0.184	Up	Up
PTGER2	NM_000956	4.44	3.79	4.11	0.050	Up	Up
PTX3	NM_002852	1.79	3.39	2.59	0.191	Up	Up
RGS4	NM_005613	1.35	2.61	1.98	0.196	Up	Up
SPOCK1	NM_004598	3.42	2.33	2.88	0.119	Up	Up
SULF1	NM_015170	7.88	5.79	6.84	0.097	Up	Up
TMEM158	NM_015444	1.65	2.62	2.14	0.142	Up	Up
TPM1	NM_000366	0.93	2.24	1.58	0.249	Up	Up
WNT5A	NM_003392	2.91	3.84	3.38	0.087	Up	Up
AGR2	NM_006408	-7.45	-8.25	-7.85	0.032	Down	Down
C10orf10	NM_007021	-1.69	-2.13	-1.91	0.073	Down	Down
CD24	L33930	-8.24	-6.81	-7.52	0.060	Down	Down
CDH1	NM_004360	-3.05	-2.69	-2.87	0.040	Down	Down
CXADR	NM_001338	-2.18	-2.56	-2.37	0.051	Down	Down
DSG3	NM_001944	-4.82	-5.18	-5.00	0.023	Down	Down
ELF3	NM_004433	-6.15	-5.35	-5.75	0.044	Down	Down
EPCAM	NM_002354	-4.51	-3.26	-3.89	0.102	Down	Down
EPHA5	BX537946	-3.55	-4.42	-3.98	0.069	Down	Down
FGFR3	NM_000142	-2.06	-2.28	-2.17	0.032	Down	Down
FST	NM_013409	-6.84	-7.98	-7.41	0.049	Down	Down
GPX3	NM_002084	-3.26	-3.32	-3.29	0.006	Down	Down
IFI30	NM_006332	-2.35	-2.11	-2.23	0.034	Down	Down
IL18	NM_001562	-6.60	-5.82	-6.21	0.040	Down	Down

KRT15	NM_002275	-1.14	-1.24	-1.19	0.027	Down	Down
LAD1	NM_005558	-8.27	-5.44	-6.86	0.130	Down	Down
MPZL2	NM_144765	-1.87	-1.74	-1.80	0.023	Down	Down
MTUS1	NM_001001927	-2.71	-3.67	-3.19	0.095	Down	Down
PKP2	NM_001005242	-3.52	-2.45	-2.98	0.113	Down	Down
PLXNB1	NM_002673	-2.58	-3.28	-2.93	0.076	Down	Down
PPL	NM_002705	-2.17	-4.00	-3.08	0.184	Down	Down
RAPGEF5	NM_012294	-1.63	-0.95	-1.29	0.164	Down	Down
RHOD	NM_014578	-6.96	-4.84	-5.90	0.113	Down	Down
S100P	NM_005980	-5.77	-8.67	-7.22	0.126	Down	Down
SERPINB1	ENST00000380739	-2.51	-2.50	-2.50	0.001	Down	Down
SLC27A2	NM_003645	-1.57	-0.96	-1.26	0.151	Down	Down
SLPI	NM_003064	-5.96	-4.24	-5.10	0.106	Down	Down
SPINT1	NM_003710	-3.46	-3.17	-3.31	0.028	Down	Down
ABCA1	NM_005502	-4.89	-4.49	-4.69	0.027	Up	Down
C5orf13	NM_004772	-2.21	-1.75	-1.98	0.074	Up	Down
CTGF	NM_001901	-5.15	-5.54	-5.35	0.023	Up	Down
DLC1	NM_182643	-1.65	-1.53	-1.59	0.024	Up	Down
FBLN1	NM_006486	-1.38	-1.13	-1.26	0.063	Up	Down
FBN1	NM_000138	-2.16	-2.19	-2.17	0.004	Up	Down
FN1	NM_054034	-4.82	-5.12	-4.97	0.019	Up	Down
GALNT10	AK021777	-1.33	-1.74	-1.54	0.085	Up	Down
IGFBP3	NM_001013398	-3.83	-4.55	-4.19	0.055	Up	Down
LOX	NM_002317	-3.65	-3.17	-3.41	0.045	Up	Down
MYL9	NM_181526	-3.43	-3.72	-3.57	0.026	Up	Down
NRP1	NM_003873	-1.47	-1.78	-1.62	0.061	Up	Down
SYT11	NM_152280	-2.00	-2.66	-2.33	0.090	Up	Down
TFPI	NM_001032281	-4.10	-4.54	-4.32	0.032	Up	Down
TGM2	NM_004613	-2.34	-3.49	-2.92	0.124	Up	Down
CDK14	NM_012395	-2.77	-1.66	-2.22	0.156	Up	Down
ADRB2	NM_000024	3.51	3.88	3.69	0.032	Down	Up
ALDH1A3	NM_000693	6.14	5.46	5.80	0.037	Down	Up
ANK3	NM_020987	1.06	1.87	1.47	0.172	Down	Up
BIK	NM_001197	1.15	4.44	2.80	0.339	Down	Up
CA2	NM_000067	4.23	4.21	4.22	0.002	Down	Up
CDS1	AK026697	0.99	1.54	1.27	0.136	Down	Up
FGFR2	NM_022970	6.28	6.08	6.18	0.010	Down	Up
KLK10	NM_002776	3.21	3.63	3.42	0.039	Down	Up
MAP7	NM_003980	6.27	7.08	6.67	0.039	Down	Up
OCLN	DQ786238	0.97	1.86	1.42	0.194	Down	Up
PLS1	NM_002670	0.92	1.78	1.35	0.196	Down	Up
ST6GALNAC2	NM_006456	1.36	1.10	1.23	0.067	Down	Up
SYK	NM_003177	8.03	8.26	8.14	0.009	Down	Up
TPD52L1	NM_001003395	2.33	2.44	2.38	0.015	Down	Up
ADAM12	NM_003474					Up	-
CDH2	NM_001792					Up	-
COL6A3	NM_057166					Up	-
FBLN5	NM_006329					Up	-
FGFR1	NM_023111					Up	-
HAS2	NM_005328					Up	-

IL1R1	NM_000877	Up	-
LTBP2	NM_000428	Up	-
LUM	NM_002345	Up	-
MME	NM_007289	Up	-
NR2F1	NM_005654	Up	-
PPAP2B	NM_003713	Up	-
PRKCA	NM_002737	Up	-
RECK	NM_021111	Up	-
SERPINE1	NM_000602	Up	-
SERPINE2	NM_006216	Up	-
SLC22A4	NM_003059	Up	-
SRGN	NM_002727	Up	-
SYNE1	NM_033071	Up	-
TAGLN	NM_001001522	Up	-
TNFAIP6	NM_007115	Up	-
TUBA1A	NM_006009	Up	-
VCAN	NM_004385	Up	-
VIM	NM_003380	Up	-
ZEB1	NM_030751	Up	-
ABLIM1	NM_001003408	Down	-
CTSL2	NM_001333	Down	-
CXCL16	NM_022059	Down	-
FAM169A	-	Down	-
FXYD3	NM_005971	Down	-
GJB3	NM_024009	Down	-
JUP	NM_002230	Down	-
KLK7	NM_005046	Down	-
KRT17	NM_000422	Down	-
LSR	NM_205834	Down	-
MBP	NM_002385	Down	-
OVOL2	NM_021220	Down	-
PRRG4	NM_024081	Down	-
PRSS8	NM_002773	Down	-
SLC7A5	NM_003486	Down	-
SMPDL3B	NM_001009568	Down	-
SORL1	NM_003105	Down	-
TMEM30B	NM_001017970	Down	-
TSPAN1	NM_005727	Down	-
ZHX2	NM_014943	Down	-
ZNF165	NM_003447	Down	-

Supplementary Table S2- Transcription factors that underwent changes during EMT

Fold change values ≥1 and ≤-1 indicate genes upregulated and downregulated in Huh7.5M cells respectively.

ZNF Family

**Gene Symbol**	**Accession Number**	**Average Fold change (log_2)**	***P* -value**
ZIC3	NM_003413	9.91	0.045
ZNF239	NM_005674	7.62	0.027
ZNF711	NM_021998	7.55	0.023
ZNF658	NM_033160	6.51	0.085
ZNF502	NM_033210	6.43	0.080
ZFPM2	NM_012082	5.82	0.042
ZEB2	NM_014795	5.26	0.009
ZNF536	NM_014717	4.54	0.064
ZNF580	NM_016202	3.91	0.105
ZNF488	NM_153034	3.27	0.090
ZNF512B	NM_020713	3.18	0.137
ZNF540	NM_152606	3.17	0.018
ZNF549	NM_153263	3.00	0.055
ZNF74	NM_003426	2.95	0.067
ZBTB47	NM_145166	2.88	0.089
ZNF790	NM_206894	2.78	0.104
ZNF449	NM_152695	2.48	0.077
ZNF783	NM_001004302	2.42	0.077
ZNF701	NM_018260	2.42	0.029
ZNF584	NM_173548	2.33	0.133
ZBTB8	NM_001040441	2.26	0.056
ZFP64	NM_199427	2.23	0.024
ZFHX4	NM_024721	2.20	0.328
ZNF438	NM_182755	2.19	0.332
ZNF771	NM_016643	2.16	0.234
ZNF275	NM_001080485	2.12	0.015
ZNF571	NM_016536	2.10	0.109
ZNF589	NM_016089	2.05	0.229
ZNF746	NM_152557	2.04	0.318
ZNF514	NM_032788	2.01	0.199
ZBTB44	NM_014155	2.00	0.177
ZNF768	NM_024671	1.98	0.121
ZNF18	NM_144680	1.97	0.082
ZNF354B	NM_058230	1.90	0.018
ZKSCAN2	NM_001012981	1.89	0.055
ZNF629	NM_001080417	1.84	0.074
ZNF295	NM_020727	1.73	0.084
ZNF688	NM_145271	1.66	0.161
ZNF585B	NM_152279	1.64	0.039
ZNF213	NM_004220	1.63	0.031
ZNF121	NM_001008727	1.62	0.045
ZNF673	NM_017776	1.60	0.032
ZNF75	NM_007131	1.60	0.095
ZNF318	NM_014345	1.51	0.034
ZNF33B	NM_006955	1.51	0.015
ZNF282	NM_003575	1.50	0.299
ZNF512	NM_032434	1.46	0.069

ZNF624	NM_020787	1.46	0.198
ZNF485	NM_145312	1.44	0.092
ZBTB22	NM_005453	1.42	0.131
ZNF26	NM_019591	1.40	0.009
ZNF585A	NM_152655	1.37	0.046
ZNF266	NM_006631	1.37	0.149
ZNF114	NM_153608	1.29	0.017
ZNF16	NM_006958	1.29	0.088
ZBTB25	NM_006977	1.27	0.042
ZNF25	NM_145011	1.25	0.238
ZSCAN20	NM_145238	1.25	0.116
ZNF200	NM_003454	1.24	0.117
ZNF569	NM_152484	1.22	0.163
ZNF70	NM_021916	1.21	0.117
ZNF187	NM_152736	1.20	0.019
ZNF37A	NM_001007094	1.17	0.089
ZBTB33	NM_006777	1.15	0.224
ZNF177	NM_003451	1.15	0.144
ZNF588	NM_016220	1.12	0.147
ZNF250	NM_021061	1.08	0.150
ZFP1	NM_153688	1.08	0.033
ZNF490	NM_020714	1.07	0.205
ZNF263	NM_005741	1.06	0.230
ZNF337	NM_015655	1.04	0.033
ZBTB10	NM_023929	1.04	0.255
ZNF554	NM_152303	1.02	0.216
ZNF41	NM_153380	1.01	0.016
ZNF14	NM_021030	-1.00	0.089
ZNF3	NM_032924	-1.00	0.097
ZNF675	NM_138330	-1.01	0.085
ZSCAN2	NM_181877	-1.02	0.025
ZNF498	NM_145115	-1.02	0.003
ZNF134	NM_003435	-1.03	0.089
ZHX2	NM_014943	-1.04	0.221
ZNF700	NM_144566	-1.04	0.237
ZNF501	NM_145044	-1.04	0.015
ZNF766	NM_001010851	-1.05	0.052
ZNF28	NM_006969	-1.06	0.009
ZNF714	NM_182515	-1.06	0.159
ZNF547	NM_173631	-1.08	0.003
ZNF606	NM_025027	-1.12	0.068
ZNF510	NM_014930	-1.12	0.151
ZNF230	NM_006300	-1.13	0.076
ZBTB46	NM_025224	-1.14	0.097
ZBTB2	NM_020861	-1.15	0.022
ZNF35	NM_003420	-1.16	0.058
ZNF431	NM_133473	-1.16	0.216
ZNF586	NM_017652	-1.18	0.086
ZNF223	NM_013361	-1.19	0.190
ZNF137	NM_003438	-1.20	0.021
ZNF519	NM_145287	-1.23	0.202
ZNF518	NM_014803	-1.24	0.064
ZNF784	NM_203374	-1.28	0.196
ZNF655	NM_001009956	-1.28	0.064
ZNF75A	NM_153028	-1.29	0.044
ZNF530	NM_020880	-1.30	0.015

ZNF467	NM_207336	-1.33	0.205
ZNF148	NM_021964	-1.37	0.104
ZNF226	NM_015919	-1.43	0.036
ZNF222	NM_013360	-1.43	0.044
ZNF572	NM_152412	-1.44	0.002
ZNF100	NM_173531	-1.46	0.099
ZNF221	NM_013359	-1.48	0.341
ZNF418	NM_133460	-1.51	0.050
ZNF551	NM_138347	-1.57	0.230
ZBTB5	NM_014872	-1.58	0.084
ZNF160	NM_198893	-1.58	0.070
ZNF264	NM_003417	-1.61	0.046
ZBTB41	NM_194314	-1.63	0.016
ZNF285A	NM_152354	-1.64	0.304
ZNF155	NM_003445	-1.77	0.278
ZNF37A	NM_001007094	-1.82	0.079
ZNF284	NM_001037813	-1.83	0.080
ZNF217	NM_006526	-1.84	0.086
ZNF625	NM_145233	-1.87	0.015
ZNF496	NM_032752	-1.88	0.178
ZMIZ1	NM_020338	-1.89	0.000
ZNF597	NM_152457	-1.93	0.005
ZNF92	NM_007139	-1.93	0.049
ZNF445	NM_181489	-2.13	0.200
ZNF117	NM_024498	-2.19	0.115
ZSCAN5	NM_024303	-2.21	0.077
ZNF444	NM_018337	-2.32	0.064
ZNF672	NM_024836	-2.33	0.003
ZNF44	NM_016264	-2.34	0.068
ZNF667	NM_022103	-2.38	0.149
ZNF85	NM_003429	-2.39	0.144
ZNF577	NM_032679	-2.43	0.048
ZNF22	NM_006963	-2.45	0.014
ZKSCAN1	NM_003439	-2.47	0.161
ZNF709	NM_152601	-2.62	0.075
ZNF660	NM_173658	-2.65	0.176
ZNF429	NM_001001415	-2.66	0.071
ZBTB7B	NM_015872	-2.69	0.022
ZNF493	NM_175910	-2.82	0.025
ZNF99	NM_001080409	-2.83	0.065
ZNF254	NM_203282	-2.85	0.085
ZSCAN12	NM_001163391	-2.93	0.195
ZNF626	NM_145297	-3.06	0.019
ZNF708	NM_021269	-3.16	0.168
ZNF600	NM_198457	-3.30	0.042
ZBTB16	NM_006006	-3.31	0.043
ZNF141	NM_003441	-3.37	0.103
ZNF669	NM_024804	-3.44	0.015
ZNF234	NM_006630	-3.52	0.042
ZNF680	NM_178558	-3.54	0.164
ZNF253	NM_021047	-3.61	0.011
ZNF229	NM_014518	-3.70	0.034
ZNF124	NM_003431	-3.82	0.005
ZBTB1	NM_014950	-3.84	0.031
ZNF20	NM_021143	-4.08	0.087
ZNF439	NM_152262	-4.09	0.004

ZNF670	NM_033213	-4.09	0.057
ZNF347	NM_032584	-4.11	0.139
ZNF630	NM_001037735	-4.24	0.089
ZNF69	NM_021915	-4.32	0.019
ZNF718	NM_001039127	-4.56	0.042
ZNF257	NM_033468	-5.06	0.008
ZNF563	NM_145276	-5.49	0.005
ZNF595	NM_182524	-5.74	0.024
ZIK1	NM_001010879	-5.97	0.049
ZNF545	NM_133466	-6.08	0.015
ZNF415	NM_018355	-6.57	0.067
ZNF671	NM_024833	-6.72	0.007
ZNF91	NM_003430	-7.12	0.033
ZNF682	NM_033196	-7.27	0.068
ZNF558	NM_144693	-7.62	0.027
ZNF544	NM_014480	-8.09	0.013
ZNF167	NM_018651	-8.48	0.074

HOX Family

Gene Symbol	Accession Number	Average Fold change (log_2)	*P*-value
HOXB6	NM_018952	11.91	0.010
HOXB3	NM_002146	9.73	0.010
HOXB5	NM_002147	9.00	0.000
HOXB2	NM_002145	8.71	0.049
HOXB9	NM_024017	8.60	0.013
HOXB8	NM_024016	7.99	0.017
HOXD9	NM_014213	7.15	0.002
HOXD10	NM_002148	6.70	0.000
HOXB13	NM_006361	6.67	0.021
HOXD13	NM_000523	6.44	0.087
HOXD11	NM_021192	5.18	0.033
HOXB4	NM_024015	5.06	0.046
HOXA5	NM_019102	4.45	0.112
HOXD3	NM_006898	3.24	0.108
HOXA6	NM_024014	2.90	0.321
HOXD8	NM_019558	2.87	0.174
HOXA10	NM_018951	2.74	0.028
HOXA1	NM_153620	2.63	0.110
HOXA11	NM_005523	2.55	0.272
HOXA13	NM_000522	2.38	0.188
HOXC13	NM_017410	2.20	0.222
HOXA9	NM_152739	2.04	0.222
HOXC9	NM_006897	1.42	0.195
HOXA2	NM_006735	1.40	0.305
HOXC8	NM_022658	-2.98	0.287
HOXD1	NM_024501	-3.54	0.014

FOX Family

Gene Symbol	Accession Number	Average Fold change (log_2)	*P*-value
FOXF1	NM_001451	7.33	0.071
FOXC1	NM_001453	6.68	0.032
FOXF2	NM_001452	6.49	0.001

FOXD1	NM_004472	5.08	0.032
FOXO4	NM_005938	4.62	0.021
FOXL1	NM_005250	3.24	0.096
FOXG1B	NM_005249	2.48	0.036
FOXH1	NM_003923	2.18	0.029
FOXE1	NM_004473	1.81	0.007
FOXP1	NM_001012505	1.09	0.006
FOXJ2	NM_018416	1.04	0.009
FOXB2	NM_001013735	-1.19	0.150
FOXI2	NM_207426	-1.37	0.178
FOXP4	NM_001012426	-1.92	0.091
FOXN2	NM_002158	-1.97	0.064
FOXN4	NM_213596	-1.97	0.224
FOXE3	NM_012186	-3.99	0.045
FOXA1	NM_004496	-4.04	0.051
FOXA3	NM_004497	-4.60	0.009
FOXJ1	NM_001454	-5.31	0.001
FOXQ1	NM_033260	-5.33	0.036
FOXA2	NM_021784	-6.74	0.048

Supplementary Table S3- Extracellular Matrix genes that underwent changes during EMT

Fold change values ≥1 and ≤-1 indicate genes upregulated and downregulated in Huh7.5M cells respectively.

Gene Symbol	Accession Number	Average Fold change (log_2)	*P* -value
TIMP1	NM_003254	8.65	0.046
SPON1	NM_006108	8.23	0.060
PLAT	NM_000930	8.19	0.074
COL13A1	NM_005203	7.36	0.049
LOXL1	NM_005576	7.11	0.072
SULF1	NM_015170	6.84	0.097
ITGA8	NM_003638	6.26	0.033
LAMC3	NM_006059	6.23	0.020
COL4A2	NM_001846	6.20	0.001
LAMA1	NM_005559	5.79	0.013
COL6A1	NM_001848	5.62	0.040
COL1A1	NM_000088	5.57	0.002
ADAM11	NM_002390	5.48	0.057
LAMA4	NM_001105208	5.34	0.014
ACAN	NM_013227	4.95	0.030
AMBN	NM_016519	4.95	0.054
ADAMTS4	NM_005099	4.68	0.048
ITGA4	NM_000885	4.50	0.043
COL4A6	NM_033641	4.38	0.038
DDR1	NM_013994	4.36	0.049
ADAMTS5	NM_007038	4.21	0.042
TNC	NM_002160	4.15	0.066
ADAMTS3	NM_014243	4.13	0.141
NID2	NM_007361	3.73	0.121
ITGA7	NM_002206	3.73	0.028
COL6A2	NM_058175	3.69	0.107
LOXL2	NM_002318	3.69	0.107
EMILIN3	NM_052846	3.58	0.092
LAMB3	NM_001017402	3.55	0.111
COL4A1	NM_001845	3.51	0.001
SMARCA2	NM_139045	3.45	0.129
COL5A1	NM_000093	3.42	0.002
SNED1	NM_001080437	3.34	0.111
COL11A2	NM_080679	3.31	0.017
ITGA9	NM_002207	3.22	0.024
ITGA2B	NM_000419	3.20	0.155
MXRA7	NM_001008529	3.12	0.046
MMP2	NM_004530	3.04	0.079
COL3A1	NM_000090	2.98	0.224
ADAMTS2	NM_021599	2.94	0.033
COL7A1	NM_000094	2.84	0.001
ADAM19	NM_033274	2.67	0.304
PCOTH	NM_001014442	2.40	0.106
LAMA3	NM_198129	2.16	0.062
COMP	NM_000095	2.15	0.129
ADAMTS13	NM_139025	2.06	0.116
LOXL3	NM_032603	1.90	0.045
ADAMTS1	NM_006988	1.83	0.213

PITRM1	NM_014889	1.80	0.037
CIB2	NM_006383	1.76	0.200
COL9A3	NM_001853	1.70	0.118
PLAUR	NM_001005377	1.60	0.105
ITGA3	NM_002204	1.58	0.276
ADAM23	NM_003812	1.51	0.211
PCOLCE	NM_002593	1.42	0.061
MMP25	NM_022468	1.35	0.144
MMP9	NM_004994	1.31	0.187
SMARCD3	NM_003078	1.27	0.157
SMARCAL1	NM_014140	1.26	0.028
COL4A5	NM_033380	1.15	0.039
LAMA2	NM_000426	1.13	0.101
ADAMTS19	NM_133638	1.09	0.049
GSG2	NM_031965	1.05	0.084
ADAMTSL1	NM_139238	-1.12	0.003
SERPINH1	NM_001235	-1.14	0.105
NID1	NM_002508	-1.17	0.153
HYAL2	NM_003773	-1.20	0.121
ITGB4BP	NM_181468	-1.21	0.186
EFEMP2	NM_016938	-1.22	0.202
P4HA1	NM_000917	-1.24	0.079
HYAL3	NM_003549	-1.28	0.206
HSPG2	NM_005529	-1.32	0.239
COL27A1	AK021957	-1.35	0.040
ADAMTSL5	NM_213604	-1.37	0.227
COL10A1	NM_000493	-1.38	0.111
ADAM9	NM_003816	-1.41	0.237
LAMC1	NM_002293	-1.43	0.011
CIB1	NM_006384	-1.46	0.057
PLOD2	NM_000935	-1.47	0.024
ADAM10	NM_001110	-1.47	0.073
EMILIN2	NM_032048	-1.52	0.168
SDC2	NM_002998	-1.55	0.061
PCOLCE2	NM_013363	-1.57	0.004
ADAMTS6	NM_197941	-1.61	0.004
SDC4	NM_002999	-1.71	0.057
ELA2A	NM_033440	-1.72	0.140
MMP11	NM_005940	-1.74	0.096
ELA3B	NM_007352	-1.75	0.157
ITGAV	NM_002210	-1.76	0.041
MMP14	NM_004995	-1.81	0.268
MEPE	NM_020203	-1.85	0.116
HS6ST3	NM_153456	-1.85	0.132
PLOD3	NM_001084	-1.88	0.097
MMP19	NM_002429	-1.89	0.217
ADAM32	NM_145004	-1.93	0.131
ITGB1	AF086249	-1.97	0.117
MMP15	NM_002428	-2.00	0.003
COL18A1	NM_030582	-2.22	0.087
ITGA1	NM_181501	-2.27	0.050
ITGA2	NM_002203	-2.36	0.115
SERPINB1	NM_030666	-2.54	0.082
MMP28	NM_024302	-2.60	0.151
HYAL1	NM_007312	-2.63	0.197
LAMB1	NM_002291	-2.70	0.042

SDC1	NM_001006946	-2.80	0.048
ITGA6	NM_000210	-2.84	0.037
LOXL4	NM_032211	-2.90	0.034
TGM2	NM_004613	-2.92	0.124
FLRT2	NM_013231	-3.03	0.017
COL12A1	NM_004370	-3.41	0.125
LOX	NM_002317	-3.41	0.045
FNDC3B	NM_022763	-3.45	0.045
FLRT3	NM_198391	-3.77	0.000
COL9A2	NM_001852	-3.80	0.066
HS3ST5	AK091074	-3.84	0.060
IBSP	NM_004967	-3.88	0.107
COL14A1	NM_021110	-4.43	0.034
COL5A2	NM_000393	-4.80	0.030
FN1	NM_054034	-4.97	0.019
COL1A2	NM_000089	-4.99	0.141
TIMP4	NM_003256	-5.21	0.048
ADAMTS12	NM_030955	-5.24	0.070
ITGAL	NM_002209	-5.47	0.033
ECM2	NM_001393	-5.56	0.128
SPON2	NM_012445	-5.91	0.035
CTHRC1	NM_138455	-6.45	0.096
SOD3	NM_003102	-6.50	0.189
RELN	NM_005045	-7.46	0.016
MEP1A	NM_005588	-10.58	0.001
COL2A1	NM_001844	-13.01	0.006

Supplementary Table S4- Regulated genes participating in Glucose, Fatty acid, Cholesterol and Bile acid Metabolism

Fold change values ≥1 and ≤-1 indicate genes upregulated and downregulated in Huh7.5M cells respectively.

Glycolysis and Gluconeogenesis

**Gene Symbol**	**Accession Number**	**Average Fold change (log_2)**	***P*-value**
HK1	NM_033500	10.2	0.002
PFKP	NM_002627	9.98	0.039
ENO2	NM_001975	3.07	0.030
PGK1	NM_000291	1.72	0.141
PFKL	NM_001002021	1.33	0.097
GCK	NM_033508	-1.16	0.060
ALDOA	NM_184041	-1.33	0.229
FBP2	NM_003837	-1.52	0.027
ALDOC	NM_005165	-1.7	0.058
PKLR	NM_000298	-1.94	0.120
HK2	NM_000189	-2.38	0.299
G6PC	NM_000151	-2.47	0.157
ENO3	NM_001976	-2.6	0.241
PC	NM_000920	-3.84	0.019
FBP1	NM_000507	-3.85	0.302
ALDOB	NM_000035	-4.17	0.166

Pyruvate Fermentation to Lactate

**Gene Symbol**	**Accession Number**	**Average Fold change (log_2)**	***P*-value**
LDHB	NM_002300	8.82	0.001
LDHC	NM_002301	5.91	0.002
LDHD	NM_153486	4.79	0.058

Glycogen Biosynthesis II (from UDP-D-Glucose)

**Gene Symbol**	**Accession Number**	**Average Fold change (log_2)**	***P*-value**
PGM5	NM_021965	3.58	0.103
UGP2	NM_006759	-1.26	0.040
PGM3	NM_015599	-1.27	0.090
GBE1	NM_000158	-1.58	0.102

Pentose Phosphate Pathway

**Gene Symbol**	**Accession Number**	**Average Fold change (log_2)**	***P*-value**
TKTL1	NM_012253	6.59	0.021
TKT	NM_001064	1.11	0.168
RPE	NM_006916	-1.14	0.056
RBKS	NM_022128	-2.05	0.008
H6PD	NM_004285	-2.39	0.134
RGN	NM_004683	-7.38	0.035

Acetyl-CoA Biosynthesis I (Pyruvate Dehydrogenase Complex)

Gene Symbol	Accession Number	Average Fold change (log_2)	*P* -value
PDK2	NM_002611	4.39	0.057
PDHB	NM_000925	2.27	0.021
PDK3	NM_005391	2.22	0.240
PDK4	NM_002612	1.65	0.031
PDHA1	NM_000284	1.51	0.215
PDK1	NM_002610	1.35	0.188
DLAT	NM_001931	-1.76	0.242

Acetate Conversion to Acetyl-CoA

Gene Symbol	Accession Number	Average Fold change (log_2)	*P* -value
ACSS1	NM_032501	8.79	0.017

Citrate cycle (TCA cycle)

Gene Symbol	Accession Number	Average Fold change (log_2)	*P* -value
OGDHL	NM_018245	3.57	0.030
MDH1B	NM_001039845	2.83	0.051
SDHA	NM_004168	1.45	0.235
SUCLA2	NM_003850	1.26	0.098
FH	NM_000143	-1.42	0.067
IDH1	NM_005896	-4.15	0.060

Oxidative phosphorylation

Gene Symbol	Accession Number	Average Fold change (log_2)	*P* -value
COX6B2	NM_144613	3.40	0.004
COX7B	ENST00000373335	2.50	0.036
COQ7	NM_016138	1.76	0.105
NDUFV3	NM_021075	1.62	0.004
COQ6	NM_182476	1.49	0.126
ATP5S	NM_015684	1.39	0.086
NDUFB10	NM_004548	1.24	0.213
NDUFC2	NM_004549	-1.07	0.030
COX4I2	NM_032609	-1.08	0.006
NDUFA13	NM_198537	-1.12	0.020
NDUFV2	NM_021074	-1.15	0.053
NDUFA2	NM_002488	-1.16	0.183
ATP5L	NM_006476	-1.19	0.140
NDUFA4	NM_002489	-1.42	0.233
NDUFV1	NM_007103	-1.42	0.082
NDUFA4	NM_002489	-1.70	0.168

Fatty Acid biosynthesis

Gene Symbol	Accession Number	Average Fold change (log_2)	*P* -value
FASN	NM_004104	-1.78	0.037

Mitochondrial L-carnitine Shuttle Pathway

Gene Symbol	Accession Number	Average Fold change ($\log_2$)	*P*-value
CPT1C	NM_152359	6.07	0.004
SLC27A1	NM_198580	3.84	0.036
ACSL6	NM_001009185	3.38	0.005
TMLHE	NM_018196	2.85	0.072
SLC27A5	NM_012254	2.83	0.004
SLC27A6	NM_001017372	1.88	0.195
ACSBG1	NM_015162	1.81	0.239
CPT1B	NM_152246	1.11	0.009
SLC27A2	NM_003645	-1.26	0.151
ACSL4	NM_004458	-2.36	0.040

Fatty Acid ß-oxidation I

Gene Symbol	Accession Number	Average Fold change ($\log_2$)	*P*-value
HSD17B4	NM_000414	-1.22	0.008
ACAA1	NM_001607	-1.24	0.087
SDS	NM_006843	-1.25	0.147
SCP2	NM_002979	-1.37	0.128
EHHADH	NM_001966	-2.13	0.191

Fatty Acid α-oxidation

Gene Symbol	Accession Number	Average Fold change ($\log_2$)	*P*-value
ALDH1A3	NM_000693	5.8	0.037
ALDH1A2	NM_170697	5.61	0.064
ALDH2	NM_000690	-1.07	0.042
ALDH3B1	NM_000694	-1.23	0.191
ALDH4A1	NM_003748	-2.24	0.010
ALDH1A1	NM_000689	-7.19	0.000

Triacylglycerol Biosynthesis

Gene Symbol	Accession Number	Average Fold change ($\log_2$)	*P*-value
MBOAT2	ENST00000354442	9.2	0.012
AGPAT4	NM_020133	8.19	0.170
AGPAT3	NM_020132	1.68	0.051
AGPAT2	NM_006412	-1.3	0.174
DBT	NM_001918	-1.55	0.212
ELOVL2	NM_017770	-1.6	0.069
ELOVL6	NM_024090	-1.74	0.040
GPAM	NM_020918	-2.55	0.037
MOGAT1	NM_058165	-2.57	0.187
DGAT2	NM_032564	-3.2	0.096
LPIN2	NM_014646	-3.62	0.013

Triacylglycerol Degradation

Gene Symbol	Accession Number	Average Fold change (log_2)	***P*** **-value**
LIPE	NM_005357	4.92	0.012
LPL	NM_000237	4.7	0.082
ABHD6	NM_020676	2.38	0.032
PNPLA2	NM_020376	1.03	0.043
PNPLA3	NM_025225	-1.93	0.183
ABHD12	NM_015600	-2.89	0.080
CES1	NM_001266	-3.23	0.305
MGLL	NM_007283	-4.82	0.049
LIPG	NM_006033	-4.87	0.022
AADAC	NM_001086	-8.48	0.121
LIPC	NM_000236	-9.25	0.156
PNPLA4	NM_004650	-9.35	0.075

Superpathway of Cholesterol Biosynthesis

Gene Symbol	Accession Number	Average Fold change (log_2)	***P*** **-value**
HSD17B7	NM_016371	-1.15	0.039
CYP51A1	NM_000786	-1.26	0.060
IDI1	NM_004508	-1.41	0.116
TM7SF2	NM_003273	-1.47	0.054
MVK	BC016140	-1.5	0.130
EBP	NM_006579	-1.53	0.190
HMGCR	NM_000859	-1.62	0.059
ACAT2	NM_005891	-1.74	0.118
FDPS	NM_002004	-2.1	0.078
DHCR24	NM_014762	-2.11	0.160
LSS	NM_001001438	-2.16	0.029
HMGCS1	NM_002130	-2.48	0.069
DHCR7	NM_001360	-2.56	0.033
MVD	NM_002461	-2.93	0.074
HMGCS2	NM_005518	-5.49	0.045

Bile Acid Biosynthesis, Neutral Pathway

Gene Symbol	Accession Number	Average Fold change (log_2)	***P*** **-value**
HSD3B7	NM_025193	-1.02	0.121
SCP2	NM_002979	-1.37	0.128
CYP27A1	NM_000784	-4.33	0.013
AKR1C3	NM_003739	-4.38	0.043

Supplementary Table S5- Genes involved in BMP pathway that underwent changes during EMT

Fold change values ≥1 and ≤-1 indicate genes upregulated and downregulated in Huh7.5M cells respectively.

Gene Symbol	Accession Number	Average Fold change (log_2)	*P* -value
MAGED1	AF217963	13.06	0.006
BMP7	NM_001719	9.62	0.017
NKX2-5	NM_004387	5.59	0.024
NOG	NM_005450	5.48	0.013
BMP6	NM_001718	4.51	0.011
BMP8A	NM_181809	4.39	0.059
PITX2	NM_153426	3.92	0.169
MAPK11	NM_002751	3.6	0.021
BMP5	NM_021073	3.03	0.023
SOSTDC1	NM_015464	3.02	0.169
ZNF423	NM_015069	2.82	0.247
BMP8B	NM_001720	2.34	0.188
PRKAG2	NM_016203	2.07	0.141
JUN	NM_002228	2.04	0.041
BMP1	NM_006129	1.95	0.097
SMAD9	NM_005905	1.93	0.111
MAPK3	NM_002746	1.87	0.209
MAPK13	NM_002754	1.62	0.230
HRAS	NM_005343	1.58	0.217
PRKAR2B	NM_002736	1.54	0.264
HOXC9	NM_006897	1.42	0.195
CREBBP	NM_004380	1.2	0.037
MRAS	NM_012219	1.13	0.076
PRKAR1A	NM_212472	-1.37	0.126
CREB1	NM_134442	-1.43	0.165
NFKB1	NM_003998	-1.98	0.282
PRKAR1B	NM_002735	-2.01	0.069
ATF2	NM_001880	-2.11	0.063
RUNX2	NM_001015051	-2.8	0.041
TLX2	NM_016170	-3.96	0.090
FST	NM_013409	-7.41	0.049

Supplementary Table S6- Genes involved in NF-κB pathway that underwent changes during EMT

Fold change values ≥1 and ≤-1 indicate genes upregulated and downregulated in Huh7.5M cells respectively.

Gene Symbol	Accession Number	Average Fold change (log_2)	*P* -value
PRKCZ	NM_002744	8.56	0.004
AKT3	ENST00000366539	8.53	0.025
FGFR2	NM_022970	6.18	0.010
CD21	NM_001006658	5.04	0.004
PRKCQ	NM_006257	4.4	0.089
FLT1	NM_002019	4.38	0.097
DDR1	NM_013994	4.36	0.049
FLT4	NM_002020	4.34	0.008
NTRK2	NM_001007097	3.08	0.050
SIGIRR	NM_021805	2.88	0.230
PIK3CD	NM_005026	2.66	0.124
CD40	NM_001250	2.43	0.115
NGFR	NM_002507	2.3	0.266
TRAF5	NM_004619	2.25	0.130
ZAP70	NM_001079	2.22	0.097
ARAF	NM_001654	2.01	0.172
IRAK1	NM_001569	1.62	0.152
HRAS	NM_005343	1.58	0.217
PLCG2	NM_002661	1.47	0.204
PKR	NM_002759	1.30	0.163
EIF2AK2	ENST00000379156	1.3	0.163
IKBKG	NM_003639	1.29	0.039
TLR5	NM_003268	1.25	0.056
CREBBP	NM_004380	1.2	0.037
MRAS	NM_012219	1.13	0.076
MAPK8	ENST00000374189	-1	0.126
AZI2	NM_022461	-1.05	0.045
NFKBIA	NM_020529	-1.27	0.097
BRAF	NM_004333	-1.29	0.215
PIK3C2B	NM_002646	-1.34	0.071
PIK3R1	NM_181523	-1.42	0.011
KDR	NM_002253	-1.52	0.278
BCL10	NM_003921	-1.53	0.209
FADD	NM_003824	-1.54	0.039
GHR	NM_000163	-1.56	0.129
MYD88	NM_002468	-1.59	0.121
MAP3K8	NM_005204	-1.64	0.154
TANK	NM_133484	-1.81	0.046
ATM	NM_000051	-1.82	0.118
RELB	NM_006509	-1.9	0.329
TGFBR3	NM_003243	-1.92	0.022
NFKB1	NM_003998	-1.98	0.282
FGFR3	NM_000142	-2.17	0.032
TNFSF13B	NM_006573	-2.23	0.115
PDGFRB	NM_002609	-2.35	0.008
MAP2K6	ENST00000359094	-2.47	0.349
MAP4K4	NM_145686	-2.47	0.003
IL1R2	NM_004633	-3.06	0.128

EGFR	NM_201283	-3.16	0.090
TGFBR2	NM_003242	-3.24	0.119
PDGFRA	NM_006206	-3.62	0.111
FGFR4	NM_213647	-4.32	0.175
TNFRSF11B	NM_002546	-4.46	0.089
LTBR	NM_002342	-4.52	0.231
IL18	NM_001562	-6.21	0.040
HVEM	NM_003820	-6.49	0.069

Supplementary Table S7- Genes involved in Wnt/β-Catenin pathway that underwent changes during EMT

Fold change values ≥1 and ≤-1 indicate genes upregulated and downregulated in Huh7.5M cells respectively.

Gene Symbol	Accession Number	Average Fold change (log_2)	*P* -value
FZD10	NM_007197	9.63	0.012
SFRP1	NM_003012	9.33	0.005
WNT3A	NM_033131	9.04	0.013
AKT3	ENST00000366539	8.53	0.025
SOX3	NM_005634	8.05	0.027
SOX8	NM_014587	6.5	0.002
WNT6	NM_006522	5.55	0.016
FZD8	NM_031866	5.42	0.092
SOX2	NM_003106	5.35	0.037
PPP2R2C	NM_020416	4.74	0.032
WNT10B	NM_003394	4.5	0.084
CDH3	NM_001793	4.2	0.052
GNAO1	ENST00000262494	3.89	0.108
CDH12	NM_004061	3.79	0.145
WNT5A	NM_003392	3.38	0.087
PPM1J	NM_005167	3.2	0.103
SOX7	NM_031439	3.16	0.077
APC2	NM_005883	3.04	0.045
FZD2	NM_001466	2.87	0.024
LEF1	NM_016269	2.85	0.101
WNT11	NM_004626	2.74	0.237
MAP4K1	NM_007181	2.74	0.003
KREMEN2	NM_172229	2.72	0.096
TLE4	NM_007005	2.71	0.005
WNT4	NM_030761	2.64	0.107
RARG	NM_000966	2.56	0.015
FZD9	NM_003508	2.24	0.101
APPL1	NM_012096	2.21	0.023
CSNK1E	NM_152221	2.14	0.046
PPP2R5B	NM_006244	2.08	0.071
PPP2R3B	NM_013239	2.05	0.063
WNT10A	NM_025216	2.04	0.181
JUN	NM_002228	2.04	0.041
PPP2R3A	NM_002718	2	0.229
WNT5B	NM_030775	2	0.048
CD44	NM_000610	2	0.003
WNT7B	NM_058238	1.99	0.027
AXIN2	NM_004655	1.97	0.141
FZD6	NM_003506	1.89	0.268
SFRP4	NM_003014	1.75	0.195
CDKN2A	NM_058197	1.69	0.070
AXIN1	NM_003502	1.45	0.134
WNT2B	NM_004185	1.3	0.167
FZD3	NM_017412	1.25	0.175
CREBBP	NM_004380	1.2	0.037
DVL1	NM_181870	1.13	0.165
KREMEN1	NM_153379	1.13	0.084
LRP6	NM_002336	-1.05	0.076

TCF3	NM_003200	-1.08	0.091
TCF7L2	ENST00000369397	-1.14	0.083
POU5F1	NM_002701	-1.21	0.091
GNAQ	NM_002072	-1.3	0.002
SOX4	NM_003107	-1.36	0.100
WNT9A	NM_003395	-1.43	0.256
LRP1	NM_002332	-1.44	0.042
PPP2R2B	NM_004576	-1.49	0.230
MDM2	NM_002392	-1.6	0.161
CSNK1A1	AF447582	-1.86	0.189
LRP5	NM_002335	-1.9	0.158
SOX13	NM_005686	-2.04	0.065
FRAT1	NM_005479	-2.11	0.042
CCND1	NM_053056	-2.41	0.041
FRZB	NM_001463	-2.56	0.001
FZD5	NM_003468	-2.57	0.055
SOX5	NM_152989	-2.8	0.043
CDH1	NM_004360	-2.87	0.040
NR5A2	NM_205860	-3.13	0.170
WIF1	NM_007191	-3.61	0.132
SOX9	NM_000346	-3.82	0.013
DKK1	NM_012242	-6.26	0.033
UBD	NM_006398	-7.51	0.169

Supplementary Table S8- List of Developmental Transcription factors that underwent changes during EMT

Fold change values ≥1 and ≤-1 indicate genes upregulated and downregulated in Huh7.5M cells respectively.

Gene Family	Gene Symbol	Accession Number	Average Fold change (log_2)	*P* -value
ATOH	*ATOH7*	NM_145178	2.99	0.022
	ATOH8	NM_032827	4.29	0.021
BARX	*BARX1*	NM_021570	8.70	0.014
	BARX2	NM_003658	3.48	0.082
BNC	*BNC1*	NM_001717	3.87	0.025
	BNC2	NM_017637	5.90	0.002
DLX	*DLX1*	NM_178120	1.79	0.083
	DLX2	NM_004405	4.95	0.001
	DLX3	NM_005220	2.91	0.014
	DLX4	NM_138281	2.94	0.037
EYA	*EYA1*	NM_000503	3.94	0.087
	EYA2	NM_172113	7.76	0.018
	EYA4	ENST00000367895	1.47	0.050
GLI	*GLI1*	NM_005269	1.71	0.174
	GLI2	NM_005270	3.01	0.055
	GLI3	NM_000168	7.22	0.055
GSC	*GSC*	NM_173849	7.32	0.086
HEY	*HEY1*	NM_012258	4.36	0.048
	HEY2	NM_012259	3.08	0.073
	HEYL	NM_014571	3.82	0.060
LBX	*LBX1*	NM_006562	2.96	0.033
LIM	*LHX1*	NM_005568	1.36	0.035
	LHX2	NM_004789	4.09	0.015
	LHX4	NM_033343	2.16	0.195
	LHX6	NM_014368	3.79	0.014
MSX	*MSX1*	NM_002448	3.57	0.044
	MSX2	NM_002449	4.21	0.072
OLIG	*OLIG1*	NM_138983	5.75	0.015
	OLIG2	NM_005806	4.37	0.022
PITX	*PITX2*	NM_153426	3.92	0.169
PKNOX	*PKNOX2*	NM_022062	6.40	0.020
SIX	*SIX1*	NM_005982	2.67	0.024
	SIX2	NM_016932	4.41	0.035
	SIX4	NM_017420	1.99	0.086
TSHZ	*TSHZ1*	NM_005786	3.25	0.102
	TSHZ2	NM_173485	2.56	0.165
	TSHZ3	NM_020856	8.35	0.027

Supplementary Table S9- Regulated genes participating in liver specific function

Fold change values ≥1 and ≤-1 indicate genes upregulated and downregulated in Huh7.5M cells respectively.

Gene Symbol	Accession Number	Average Fold change (log_2)	*P* -value
SLC16A2	NM_006517	10.83	0.010
ABCG4	NM_022169	7.66	0.000
SLCO4A1	NM_016354	7.65	0.029
SLC6A15	NM_182767	6.98	0.044
SLC35F2	NM_017515	6.88	0.046
SLC13A3	NM_001011554	6.62	0.077
ABCC8	NM_000352	6.53	0.033
APOBEC3C	NM_014508	6.44	0.040
APOBEC3F	NM_145298	6.24	0.025
SLC7A8	NM_182728	5.78	0.051
SLC35F1	NM_001029858	5.71	0.016
SLC9A5	NM_004594	5.4	0.011
SLC13A4	NM_012450	5.35	0.035
SLC9A9	NM_173653	5.34	0.049
SLC8A1	NM_021097	5.16	0.059
ABCG1	NM_207630	5.04	0.102
CR2	NM_001006658	5.04	0.004
SLC7A3	NM_032803	4.85	0.099
SLC25A12	NM_003705	4.84	0.020
APOOL	NM_198450	4.73	0.102
SLC4A3	NM_005070	4.64	0.036
CYP4F22	NM_173483	4.46	0.082
ITIH5	NM_030569	4.43	0.073
CYP2S1	NM_030622	4.01	0.023
SLC32A1	NM_080552	3.94	0.197
CYP2E1	NM_000773	3.85	0.063
SLC27A1	NM_198580	3.84	0.036
SLC35F3	NM_173508	3.79	0.044
SLCO3A1	NM_013272	3.79	0.043
SLC22A17	NM_016609	3.55	0.070
C1QL4	NM_001008223	3.49	0.082
SLC16A14	NM_152527	3.49	0.065
APOBEC3D	NM_152426	3.46	0.159
SLC6A8	NM_005629	3.42	0.050
SLC24A4	NM_153646	3.29	0.100
CYP27C1	NM_001001665	3.24	0.033
CYP27B1	NM_000785	3.22	0.136
SLC25A35	NM_201520	3.22	0.075
SLCO5A1	NM_030958	3.14	0.028
C1QL4	NM_001008223	2.99	0.078
SLC25A12	NM_003705	2.98	0.031
SLC2A3	NM_006931	2.97	0.052
SLC27A5	NM_012254	2.83	0.004
SLC7A1	NM_003045	2.76	0.055
CYP2U1	NM_183075	2.7	0.066
SLC2A10	NM_030777	2.56	0.082
ITIH4	NM_002218	2.47	0.085
CYP26A1	NM_057157	2.38	0.304
SLC2A12	NM_145176	2.35	0.034

CFHR4	NM_006684	2.32	0.131
SLC9A7	NM_032591	2.3	0.003
APOBEC3B	NM_004900	2.29	0.258
CYP4X1	NM_178033	2.28	0.078
SLC12A5	NM_020708	2.22	0.074
SLC30A3	NM_003459	2.19	0.207
C1QL1	NM_006688	2.17	0.054
CD55	NM_000574	2.12	0.091
SLC25A41	NM_173637	2.08	0.152
SLC25A14	NM_003951	2.07	0.150
C1R	NM_001733	2.04	0.249
C13orf15	NM_014059	2.02	0.013
SLC44A2	NM_020428	1.94	0.043
FANCE	NM_021922	1.93	0.059
UBE1	NM_003334	1.92	0.076
SLC41A1	NM_173854	1.89	0.008
SLC27A6	NM_001017372	1.88	0.195
SLC25A26	NM_173471	1.85	0.050
SLC22A5	NM_003060	1.78	0.128
CYP1B1	NM_000104	1.7	0.300
SLC9A6	NM_006359	1.7	0.152
FANCC	NM_000136	1.67	0.109
SLC25A6	NM_001636	1.62	0.035
SLC6A9	NM_201649	1.6	0.242
SLC26A2	NM_000112	1.59	0.149
SLC12A6	NM_005135	1.58	0.098
ABCC1	NM_019862	1.51	0.119
SLC10A4	NM_152679	1.5	0.253
CYP4V2	NM_207352	1.49	0.081
SLC25A17	NM_006358	1.43	0.051
SLC25A21	NM_030631	1.42	0.061
SLC25A4	NM_001151	1.42	0.111
MT1F	NM_005949	1.39	0.240
SLC25A11	NM_003562	1.36	0.016
ERCC6	NM_000124	1.33	0.069
APOO	NM_024122	1.32	0.072
ABCB9	NM_019625	1.24	0.134
SLC35B4	NM_032826	1.22	0.139
SERPINB6	NM_004568	1.17	0.043
SLC9A1	NM_003047	1.13	0.011
SLC25A23	NM_024103	1.09	0.026
APOLD1	NM_030817	1.08	0.119
APOL2	NM_145637	1.07	0.112
SLC30A4	NM_013309	1.02	0.059
SLC25A34	NM_207348	-1.02	0.111
XPC	NM_001145769	-1.02	0.081
SLC19A2	NM_006996	-1.06	0.015
SLC22A12	NM_144585	-1.07	0.080
SLC17A5	NM_012434	-1.1	0.133
SLC38A2	NM_018976	-1.1	0.151
SLC5A6	NM_021095	-1.1	0.128
SERPINH1	NM_001235	-1.14	0.105
SLC35A2	NM_001032289	-1.14	0.031
APOBEC4	NM_203454	-1.17	0.030
SLC2A13	NM_052885	-1.17	0.148
CYP51A1	NM_000786	-1.19	0.011

SLC46A1	NM_080669	-1.21	0.198
CYP2W1	NM_017781	-1.23	0.057
SLC5A12	NM_001042366	-1.25	0.131
SLC27A2	NM_003645	-1.26	0.151
GOT1	NM_002079	-1.27	0.133
SLC7A6OS	NM_032178	-1.27	0.138
SLC29A3	NM_018344	-1.28	0.064
CYP2J2	NM_000775	-1.3	0.128
SLC38A6	NM_153811	-1.32	0.145
C1QL2	NM_182528	-1.34	0.122
SLC6A16	NM_014037	-1.37	0.112
PLGLB1	NM_001032392	-1.38	0.030
SLC22A20	NM_001004326	-1.38	0.191
APOB48R	NM_018690	-1.4	0.048
RBPMS	NM_001008712	-1.43	0.062
SLC2A4RG	NM_020062	-1.43	0.018
CD46	NM_002389	-1.48	0.039
SLC30A7	NM_133496	-1.49	0.006
TFR2	NM_003227	-1.52	0.027
SLC23A2	NM_203327	-1.56	0.117
SLC25A42	NM_178526	-1.56	0.020
GPT	NM_005309	-1.59	0.246
SLC12A4	NM_005072	-1.61	0.104
C7	NM_000587	-1.66	0.067
CYP26B1	NM_019885	-1.67	0.038
SLC22A16	NM_033125	-1.69	0.190
ALDOC	NM_005165	-1.7	0.058
SLC12A2	NM_001046	-1.73	0.171
MET	NM_000245	-1.74	0.015
SLC20A2	NM_006749	-1.76	0.058
APOA5	NM_052968	-1.78	0.218
ABCA4	NM_000350	-1.8	0.035
ATM	NM_000051	-1.82	0.118
SLC38A3	NM_006841	-1.82	0.236
SLC4A4	NM_003759	-1.84	0.040
RRBP1	NM_004587	-1.91	0.173
SLC6A11	NM_014229	-1.91	0.328
SLC2A4	NM_001042	-1.92	0.045
SLC25A20	NM_000387	-1.93	0.041
ABCD3	NM_002858	-1.94	0.099
SERPINB9	NM_004155	-1.96	0.071
SLC9A3R1	NM_004252	-2.03	0.079
HNF4G	NM_004133	-2.04	0.130
PLGLB2	NM_002665	-2.05	0.054
SLC7A2	NM_003046	-2.05	0.120
SLC39A14	NM_015359	-2.12	0.006
ABCA10	NM_080282	-2.14	0.025
HDGF	NM_004494	-2.14	0.099
RBP5	NM_031491	-2.14	0.153
ABCB6	NM_005689	-2.15	0.049
SLC4A7	NM_003615	-2.15	0.031
APOA4	NM_000482	-2.16	0.170
APOC4	NM_001646	-2.17	0.225
SLC2A8	NM_014580	-2.17	0.187
CYP20A1	NM_177538	-2.24	0.076
SLC22A18AS	NM_007105	-2.28	0.011

C1S	NM_001734	-2.29	0.253
HNF4A	NM_178849	-2.31	0.075
FTH1	NM_002032	-2.37	0.015
SLC22A18	NM_183233	-2.43	0.149
SLC25A13	NM_014251	-2.56	0.025
SLC17A3	NM_006632	-2.6	0.229
ONECUT2	NM_004852	-2.63	0.022
SLC44A3	NM_152369	-2.67	0.208
SLC26A1	NM_022042	-2.7	0.005
ABCB1	NM_000927	-2.73	0.025
SLC44A1	NM_080546	-2.74	0.035
SLC6A13	NM_016615	-2.76	0.045
SLCO4C1	NM_180991	-2.81	0.019
CYP1A1	NM_000499	-2.86	0.328
SLC3A1	NM_000341	-2.88	0.229
SLC6A12	NM_003044	-2.93	0.187
AGTR1	NM_031850	-2.96	0.072
SLC23A1	NM_152685	-2.99	0.152
ABCG2	NM_004827	-3.11	0.085
ABCC6	NM_001171	-3.14	0.020
ABCB4	NM_018850	-3.15	0.094
SLCO1B1	NM_006446	-3.15	0.148
CYP3A5	NM_000777	-3.17	0.084
MT1M	NM_176870	-3.2	0.160
PON2	NM_000305	-3.24	0.026
SERPINA7	NM_000354	-3.25	0.023
MST1	NM_020998	-3.28	0.139
FOXA3	NM_004497	-3.3	0.002
FTL	NM_000146	-3.32	0.017
SLC7A7	NM_003982	-3.38	0.012
SLC29A2	NM_001532	-3.39	0.105
SLC29A4	NM_153247	-3.39	0.013
CYP2B6	NM_000767	-3.43	0.170
RBP7	NM_052960	-3.44	0.152
SLC6A6	NM_003043	-3.44	0.033
CYP3A7	NM_000765	-3.65	0.011
SLC17A1	NM_005074	-3.7	0.006
SLC12A8	NM_024628	-3.81	0.035
SLC1A7	NM_006671	-3.82	0.142
CFHR1	NM_002113	-3.88	0.051
APOC1	NM_001645	-3.89	0.040
CFHR3	NM_021023	-3.98	0.261
SERPINA4	NM_006215	-3.98	0.054
SLC16A5	NM_004695	-3.99	0.028
FOXA1	NM_004496	-4.04	0.051
SERPINA10	NM_016186	-4.07	0.170
ITIH1	NM_002215	-4.1	0.099
ALDOB	NM_000035	-4.17	0.166
HLF	NM_002126	-4.22	0.011
SERPING1	NM_000062	-4.29	0.044
SLC17A2	NM_005835	-4.29	0.007
CYP27A1	NM_000784	-4.33	0.013
SLC40A1	NM_014585	-4.41	0.020
ITIH3	NM_002217	-4.46	0.107
SLCO1A2	NM_005075	-4.5	0.071
ABCA1	NM_005502	-4.69	0.027

C8B	NM_000066	-4.73	0.030
CFB	NM_001710	-4.75	0.081
CYP2C18	NM_000772	-4.79	0.024
ONECUT1	NM_004498	-4.84	0.016
SEPP1	NM_001085486	-4.89	0.046
CFI	NM_000204	-4.94	0.019
FN1	NM_054034	-4.97	0.019
SLC30A10	NM_018713	-4.98	0.022
CYP4F2	NM_001082	-4.99	0.052
CYP2C9	NM_000771	-5.04	0.043
SLC43A3	NM_199329	-5.05	0.002
SLC38A4	NM_018018	-5.25	0.011
SERPINA3	NM_001085	-5.28	0.044
SLC16A7	NM_004731	-5.3	0.006
C2	NM_000063	-5.38	0.028
APOM	NM_019101	-5.45	0.005
SLC2A2	NM_000340	-5.45	0.130
SLC25A18	NM_031481	-5.62	0.008
C1RL	NM_016546	-5.66	0.055
CP	NM_000096	-5.66	0.084
SLC5A9	NM_001011547	-5.89	0.038
FGA	NM_021871	-5.92	0.010
C4BPB	NM_000716	-5.93	0.176
APOB	NM_000384	-5.98	0.316
TCF2	NM_000458	-6.02	0.019
SLC26A3	NM_000111	-6.04	0.101
SLC6A4	NM_001045	-6.06	0.079
SLC22A9	NM_080866	-6.07	0.015
ABCC3	NM_003786	-6.08	0.121
SERPINF2	NM_000934	-6.09	0.081
SLC6A14	NM_007231	-6.26	0.051
SLC22A3	NM_021977	-6.34	0.105
KNG1	NM_000893	-6.36	0.049
ABCC2	NM_000392	-6.43	0.043
FOXA2	NM_021784	-6.74	0.048
SLC39A5	NM_173596	-6.79	0.018
CYP4F12	NM_023944	-7.22	0.033
PLG	NM_000301	-7.29	0.142
HPX	NM_000613	-7.35	0.002
ALB	NM_000477	-7.65	0.006
CYP4F8	NM_007253	-7.68	0.033
PON1	NM_000446	-7.75	0.024
VTN	NM_000638	-7.8	0.030
CFH	NM_001014975	-7.96	0.055
C5	NM_001735	-8.18	0.056
PON3	NM_000940	-8.31	0.018
RBP2	NM_004164	-8.35	0.041
C4BPA	NM_000715	-8.48	0.086
SLCO1B3	NM_019844	-8.48	0.005
SLCO2B1	NM_007256	-8.54	0.017
SLC16A3	NM_004207	-8.77	0.045
SERPINA6	NM_001756	-8.85	0.030
C8A	NM_000562	-8.88	0.063
APOC3	NM_000040	-9.16	0.075
LIPC	NM_000236	-9.25	0.156
SLC13A5	NM_177550	-9.29	0.011

SLC19A3	NM_025243	-9.36	0.023
HP	NM_005143	-9.38	0.088
SLC35D2	NM_007001	-9.49	0.022
SERPINA5	NM_000624	-9.62	0.024
SLC7A10	NM_019849	-9.68	0.013
APOE	NM_000041	-9.76	0.017
ACF	NM_138933	-9.88	0.024
SERPINC1	NM_000488	-10.12	0.039
SERPIND1	NM_000185	-10.2	0.016
C3	NM_000064	-10.31	0.023
ITIH2	NM_002216	-10.34	0.028
ORM1	NM_000607	-10.51	0.061
FGG	NM_000509	-10.53	0.051
ORM2	NM_000608	-10.55	0.099
TCF1	NM_000545	-11.03	0.028
AHSG	NM_001622	-11.1	0.007
APOC2	NM_000483	-11.13	0.008
ARG1	NM_000045	-11.29	0.026
SERPINA1	NM_001002236	-11.3	0.014
FGB	NM_005141	-11.58	0.087
FGL1	NM_201553	-12	0.028
AGT	NM_000029	-12.14	0.066
AFP	NM_001134	-12.36	0.008
FABP1	NM_001443	-12.38	0.044
APOA1	NM_000039	-12.44	0.012
APOH	NM_000042	-12.59	0.033
TTR	NM_000371	-12.6	0.019
AMBP	NM_001633	-13.4	0.052
APOA2	NM_001643	-13.43	0.004
RBP4	NM_006744	-13.59	0.007

Supplementary Table S10- List of primers used for microarray validation

Primer Name	Sequence (5' – 3')
EMP3 For	CTCTGCTGTCTCTCCTTCATCCTG
EMP3 Rev	AGATCAAGGCGCCAGTAAACAC
IL18 For	TGACCAAGGAAATCGGCCTCTAT
IL18 Rev	GCCATACCTCTAGGCTGGCTATCT
FSTL1 For	ACGATGGACACTGCAAAGAGAAGA
FSTL1 Rev	AACCAGCCATCTGGAATGATCTC
SYT11 For	GACAGTGGTGGTCCTCAAAGCC
SYT11 Rev	TTCTTCTTGGCAATGCGCTTTCT
ALDH1A3 For	GGAGTATGCCAAGAAACGGCC
ALDH1A3 Rev	CCTTCCTTCTTCCCACTCTCGATC
ABCA1 For	CAGACGGAGTCGGAAAGGATTTT
ABCA1 Rev	AGTTCCAGGCTGGGGTACTT
CTGF For	GGCCTCTTCTGTGACTTCGGCTC
CTGF Rev	GTCCAGGCACGTGCACTGGTACTTG
FN1 For	TACCTAGGCAATGCGTTGGTTTGTAC
FN1 Rev	CCAGATCATGGAGTCTTTAGGACGCTCAT
WNT5A For	TTCTCCTTCGCCCAGGTTGTAATTGAAG
WNT5A Rev	TCCTGATACAAGTGGCACAGTTTCTTCTG
LAD1 For	AATGGAGACCGGCAGGCCTCTGCTT
LAD1 Rev	TGCCGTGTTCTGAGGATGCTCTGG
COL1A1 For	CCTGGCCGAGATGGCATCCCTG
COL1A1 Rev	TTGATTTCTCATCATAGCCATAAGACAGCTGG
ACSS1 For	AGAGCGTTGCTTTGATCTGG
ACSS1 Rev	GGTGGACTCCATGCCTCTT
MDH1B For	ACACAACGTCCTGAGGTTTGG
MDH1B Rev	CAGCTCTCTCCAGATGATAGGG
SUCLA2 For	GTGAGCGAAAATATCCCAGG
SUCLA2 Rev	TCTTCAATGTTGACACCACCA
HMGCR For	TTCGGTGGCCTCTAGTGAGA
HMGCR Rev	TGTCCCCACTATGACTTCCC
HMGCS2 For	CCTCTTCAATGCTGCCAACT
HMGCS2 Rev	ACCTGTGGGACGAGCATTAC
FASN For	GCTCCAGCCTCGCTCTC

FASN Rev	AACTCCTGCAAGTTCTCCGA
LIPC For	ATCAAGTGCCCTTGGACAAAG
LIPC Rev	TCCAAAGAGCAGGAATCTGG
UGP2 For	AAAAATCCAGAGACCCCCTG
UGP2 Rev	GTTCCCAAACCACCATTGAG
GBE1 For	CCAGACTCCTGGAGATCGAC
GBE1 Rev	CCACCTTCATTTTCTCCAATG
ALB For	AGCTCGGCTTATTCCAGGGG
ALB Rev	CATGATCTTCAAATGGACACTGCTG
GOT1 For	GGCACCTCCGTCAGTCTTTG
GOT1 Rev	GGCTATTGTCATTAGCAATCTTCTGC
GPT For	CTTGGAGCTGGAGCAGGAGCTGCG
GPT Rev	GGCTGCTCAGAAGATCAGGGTTAACAC
AFP For	AATCCAGAACACTGCATAGAAATGAATATGG
AFP Rev	CCTGAAGACTGTTCATCTCCAGTGGG
HNF1A For	ATGAAGACGCAGAAGCGGGCCG
HNF1A Rev	ACTTGAAACGGTTCCTCCGCCC
HNF4A For	GCGGAAGAACCACATGTACTCCTG
HNF4A Rev	CATAGCTTGACCTTCGAGTGCTGA

Supplementary Table S11- List of primers used for overexpression studies

Primer Name	Sequence (5' – 3')
HA-β-Catenin For	CGGGGTACCGCCACCATGTACCCATACGATGTTCCA GATTACGCTATGGCTACTCAAGCTGATTTG
β-Catenin Rev	CGCGGATCCTTACAGGTCAGTATCAAACCAGGC
HA-GSK-3β For	GGGGTACCGCCACCATGTACCCATACGATGTTCCAG ATTACGCTATGTCAGGGCGGCCCAGAAC
GSK-3β Rev	GCTCTAGATCAGGTGGAGTTGGAAGCTGATGCAGAA GCAGCATTATTGG
GSK-3β K85A KD For	CTGGTCGCCATCGCGAAAGTATTGCAG
GSK-3β K85A KD Rev	CTGCAATACTTTCGCGATGGCGACCAG
